# Inflammatory Exposure and Depression in Older Adults With Insomnia: A Randomized Clinical Trial

**DOI:** 10.1001/jamapsychiatry.2025.1327

**Published:** 2025-07-16

**Authors:** Michael R. Irwin, Chloe C. Boyle, Joshua H. Cho, Dominique Piber, Nina Sadeghi, Daisy Castillo, Michael T. Smith, Naomi I. Eisenberger, Richard Olmstead

**Affiliations:** 1 Cousins Center for Psychoneuroimmunology, Jane and Terry Semel Institute for Neuroscience and Human Behavior, University of California, Los Angeles; 2 Department of Psychiatry and Biobehavioral Sciences, David Geffen School of Medicine, University of California, Los Angeles; 3 Department of Psychiatry and Neurosciences, Charité–Universitätsmedizin Berlin, Berlin, Germany; 4 Corporate Member of Freie Universität Berlin, Humboldt-Universität zu Berlin, Berlin, Germany; 5 Berlin Institute of Health, Berlin, Germany; 6 Department of Psychiatry and Behavioral Sciences, School of Medicine, Johns Hopkins University, Baltimore, Maryland; 7 Department of Psychology, College of Arts and Sciences, University of California, Los Angeles

## Abstract

**Importance:**

Insomnia and inflammation are prevalent in older adults, and both are risk factors for late-life depression. Older adults with insomnia who are exposed to inflammatory challenge may be more vulnerable to depression.

**Objective:**

To determine whether inflammatory exposure induces greater increases in depressive mood and symptoms in older adults with insomnia disorder compared to those without insomnia.

**Design, Setting, and Participants:**

This assessor-blinded, parallel-condition randomized clinical trial was conducted from August 2017 to November 2022 at a single site in Los Angeles, California, among a community-based sample of 160 nondepressed adults aged 60 years or older (53 with insomnia disorder and 107 without insomnia, or control). Data analysis occurred from July 2023 to August 2024.

**Interventions:**

Participant groups stratified by insomnia status were randomized to 2 conditions: endotoxin or placebo.

**Main Outcomes and Measures:**

The primary outcome was depressed mood, assessed by the Profiles of Mood States depression subscale (POMS-D). Secondary outcomes were depressive symptom severity and inflammatory cytokines.

**Results:**

Among 160 randomized participants eligible for the study (mean [SD] age, 65.9 [4.6] years; 84 female participants [52.5%]), 79 participants (26 with insomnia, 53 control participants) were randomized to endotoxin and 81 (27 with insomnia, 54 control participants) to placebo. All randomized participants completed the protocol. Compared to placebo, endotoxin induced increases in POMS-D to a significantly greater extent in those with insomnia than controls (condition × group interaction, *F*_10,1478_ = 4.7; *P* < .001), with a similar effect for observer-rated POMS-D mood (condition × group interaction, *F*_3,450_ = 5.5; *P* = .001), as well as clinically meaningful increases in observer-rated measures of depressive symptoms. Endotoxin induced similar increases in inflammatory cytokines in both groups. Moderation analyses found that the inflammatory response was associated with increases in POMS-D in the insomnia group (β = 0.33; 95% CI, 0.26-0.41; *P* < .001) but not in control participants.

**Conclusions and Relevance:**

In this randomized clinical trial, older adults with insomnia showed an exaggerated vulnerability to depressive mood and symptoms in response to inflammatory challenge. Older adults with insomnia should undergo vigilant depression monitoring during periods of inflammatory exposure; selective depression prevention strategies that target both insomnia and inflammatory phenotypes are needed.

**Trial Registration:**

ClinicalTrials.gov Identifier: NCT03256760

## Key Points

**Question:**

Does inflammatory exposure lead to exaggerated increases in depressive symptoms in adults aged 60 years or older with insomnia?

**Findings:**

In this randomized clinical of 160 adults aged 60 years or older, experimental induction of inflammatory activation by administration of endotoxin led to a significant, 3-fold greater increase in depressive mood symptoms in those with insomnia compared to those without insomnia. Increases in depressive responses were more persistent in participants with insomnia.

**Meaning:**

Older adults with insomnia show a heightened vulnerability to depressed mood and subthreshold depressive symptoms following inflammatory exposure.

## Teaser

This parallel-condition randomized clinical trial determines whether inflammatory exposure induces greater increases in depressive mood and symptoms in older adults with insomnia disorder compared to those without insomnia.

## Introduction

Major depressive disorder in adults 60 years and older shows a 12-month prevalence that exceeds 10%^1^ and contributes to cognitive decline, disability, medical comorbidity, and all-cause mortality.^2,3^ Further, older adults are likely to experience chronic inflammation (ie, inflammaging).^4,5,6^ On a behavioral level, insomnia is causally tied to depression risk^7,8^ and its prevention,^9^ whereas on the biological level, inflammation is increasingly viewed as contributing to some types of depression.^10,11^ However, to our knowledge, no study has tested whether older adults with insomnia are more vulnerable to symptoms of depression in response to inflammatory exposure. Such experimental substantiation would constitute evidence of a mechanistic link between insomnia, inflammation, and depression and would demonstrate the salience of inflammatory exposure in precipitating depressive symptoms in older adults with insomnia.

Here, we present results from a randomized clinical trial designed to test whether older adults with insomnia display greater increases in depressive mood and symptoms in response to endotoxin compared to older adults without insomnia. Endotoxin challenge is a reliable, safe, and well-validated method of transiently inducing robust peripheral and central nervous system (CNS) inflammation^12,13,14,15^ and causing increases in depressive symptoms in healthy adults.^12,16,17^ We hypothesize that administration of endotoxin (0.8 ng/kg body weight) vs placebo will induce greater increases in self- and observer-rated assessment of depressed mood (primary outcome) and observer-rated clinical measures of depressive symptoms (secondary outcome) in older adults with insomnia compared to those without insomnia.

## Methods

### Trial Design and Oversight

This double-blinded, parallel-design, single-site randomized clinical trial adhered to the Consolidated Standards of Reporting Trials (CONSORT) reporting guidelines. Following voluntary written informed consent, as approved by the University of California, Los Angeles (UCLA), institutional review board (16-000583), participants were enrolled between August 22, 2017, and November 20, 2022. The trial protocol was conducted in accordance with the Declaration of Helsinki, prospectively registered on ClinicalTrials.gov (NCT03256760), reviewed by independent trial data monitoring and steering committees of the UCLA Clinical and Translational Science Institute (CTSI), and is available in Supplement 1.^18^ A US National Institutes of Health (NIH)–mandated data and safety monitoring board provided oversight.

### Participants

A community sample of older adults 60 to 80 years old were identified using a database of households with at least 1 person older than 60 years within 15 miles of UCLA.^19,20^ Further, we queried electronic health records at UCLA Health, which were linked to older adult legacy systems. Participants were contacted by brochure, with follow-up phone contact.^9,21,22,23,24^

After screening eligibility, interviews confirmed the presence or absence of insomnia disorder,^25,26^ absence of major depressive disorder in the last year, and absence of a circadian rhythm sleep-wake disorder confirmed by sleep diary information. Full eligibility criteria are described in Supplement 1.^18^ Self-reported race and ethnicity data informed generalizability and satisfied NIH requirements. Approximately twice as many control participants vs insomnia participants were recruited, given feasibility and cost utility, yielding greater statistical power.

### Trial Procedures

Participants were randomly allocated to 2 conditions—endotoxin, 0.8 ng per kg body weight, or placebo condition—in a 1:1 ratio, stratified by insomnia. Pretreatment classification variables (age, 60-70 or 71-80 years; sex; body mass index [BMI, calculated as weight in kilograms divided by height in meters squared], 18-24.9 or 25-35) were characterized and used in a modified randomization procedure (minimization method)^27^ to ensure that group and condition were balanced. Randomization was performed using computer-generated random number sequence; allocation concealment was maintained by using an encrypted email to the UCLA CTSI pharmacy. Participants were blinded to allocation condition and outcomes; assessors were blinded to condition. No instances of unblinding occurred.

The NIH provided reference endotoxin for humans (*Escherichia coli* group O:113) as previously administered^28^; placebo was saline. Low-dose endotoxin mimics increases in inflammatory cytokines, interleukin 6 (IL-6), and tumor necrosis factor α (TNF), as found in chronic inflammatory conditions or infectious exposure,^13,29^ and induces reproducible and robust effects on depression responses.^12^ Two other models have been used to evaluate the association between inflammation and depression: interferon alfa (IFN-a) increases depressive symptoms after 12 weeks but cannot ethically be given to healthy volunteers,^12,30^ while typhoid vaccination induces modest increases in inflammation and minimal changes in depressed mood.^31,32^

After administration of endotoxin vs placebo, vital signs and blood sampling were obtained every half hour for the first hour and then hourly for up to 9 hours, with primary and secondary outcomes repeatedly assessed as described.^18^ Once safety of endotoxin in older adults was confirmed (n = 20), the protocol was modified from 12 to 9 hours’ duration to reduce participant burden.

### Outcomes

The primary outcome was self- and observer-rated assessment of depressed mood as measured by the Profiles of Mood States depression subscale (POMS-D).^33,34,35^ POMS-D was completed at baseline, 30 minutes, and then hourly, and observer-rated POMS-D was completed at baseline and every 2 hours.

To provide a comprehensive assessment of depression responses, observer-rated clinical measures (Montgomery-Åsberg Depression Rating Scale [MADRS]^36^; Hamilton Rating Scale for Depression [HAMD],^37,38^ modified with omission of insomnia items) were obtained every 2 hours, along with depression-related exploratory outcomes (feelings of social disconnection^39^; MADRS anhedonia item). Circulating levels of IL-6 and TNF were assayed as described^28,40^ (eMethods in Supplement 2). “Sickness symptoms” (eg, headache, muscle pain, shivering, nausea, breathing difficulties) were evaluated with the Physical Symptom Questionnaire,^16,41^ with dichotomized scoring (≥3) as a severe response (Supplement 1).

### Adverse Events

Adverse events were actively monitored during the protocol by vital signs and sickness symptoms. Furthermore, participants underwent a phone interview 1 day and 7 days after the protocol, with assessment of physical and depressive symptoms.

### Sample Size

Low-dose endotoxin induces increases in POMS-D with moderate effects (*d*, 0.48-0.65)^16,41,42^ and with a large effect (*d* = 0.71) in young female adults with mild sleep disturbance (insomnia disorder).^43^ Monte Carlo simulations indicate that sample sizes ranging from 42 to 60 yield a minimum of 85% power (2-tailed α = .05) for main effect of condition (ie, endotoxin vs placebo), main effect of group (ie, insomnia vs comparison controls), and within-time comparisons by group and by condition in which omnibus linear mixed models (LMM) analysis of repeated measures yield greater statistical power (>90%). However, the large group effect was estimated from a single small study.^16^ To assure statistical power, controls were oversampled (n = 100), yielding greater than 90% power for main and interaction effects.

### Statistical Analysis

Analyses were reported according to the CONSORT statement,^44^ prespecified in the statistical analysis plan without modification^18^ (Supplement 1), and undertaken according to the intention-to-treat principle.

The primary outcome—POMS-D—was analyzed with LMM with main effects of group, condition, and group by condition; interaction with time variable was reported. Group and condition are between-participants factors with repeated measures within participants; covariance structure for repeated measures was assumed as compound symmetry. Alternate covariance structures (ie, unstructured, autoregressive 1, and Toeplitz) yielded similar results. Age, sex, and BMI are related to depression responses to endotoxin^16,45,46^; hence, analyses covaried for age, sex, and BMI. Secondary outcomes were also analyzed with adjusted LMM. Further analyses evaluated the mean difference in the area under the curve (MD-AUC) between endotoxin vs placebo for each group, followed by within-time comparisons for each time point (for example, T_2_ refers to the 2-hour time point, etc). Levels of IL-6 and TNF were natural log-transformed to achieve normalization, which was not needed for other outcomes.^16^ An additive inflammatory composite was created for mediation and moderator analyses. Significance was defined by *P* < .05.

Predefined sensitivity analysis examined effects as a function of sex. Within the endotoxin condition, sensitivity analyses estimated group difference and subgroup differences.

Planned exploratory analyses examined the relation between inflammatory responses and the primary outcome within the endotoxin condition. First, using the methods of Hayes and colleagues and the Hayes PROCESS macro (version 4.1),^47,48,49,50^ we tested whether the endotoxin effect on POMS-D was mediated by the inflammatory response. Second, using a mixed-effects regression model, we tested whether the effect of the inflammatory response on POMS-D was moderated by group.

IBM SPSS version 30 (IBM) was used for all analyses.

## Results

### Participants

**Figure 1.**
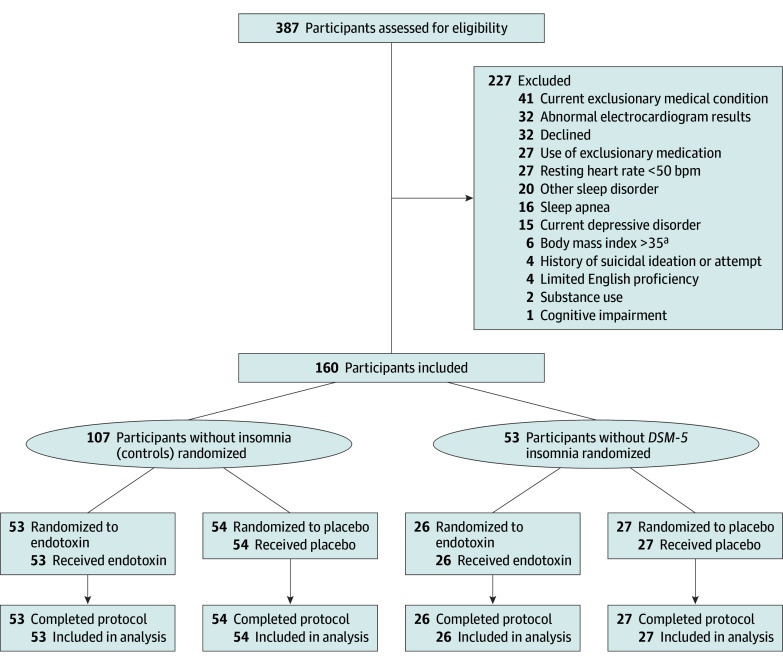
Eligibility Enrollment, Randomization, and Assessment Details regarding screening, eligibility assessment, treatment delivery, and follow-up evaluation are found in the Methods and in the trial protocol (Supplement 1). ^a^Calculated as weight in kilograms divided by height in meters squared.

A total of 387 individuals underwent eligibility evaluation. Among those eligible (N = 160; mean [SD] age, 65.9 [4.6] years; 84 female participants [52.5%]; 19 African American/Black [11.9%], 12 Asian [7.5%], 11 Hispanic [6.9%], 5 multiracial [3.1%], 3 Pacific Islander [1.9%], and 121 White [75.6%] participants), 79 participants (26 insomnia, 53 control) were randomized to endotoxin and 81 (27 insomnia, 54 control) to placebo (Figure 1).

Groups showed similar clinical characteristics, except that insomnia and depressive symptoms and depression history were greater in the insomnia group (Table). Groups by randomization condition showed balanced characteristics (eTable 1 in Supplement 2).

**Table.** Characteristics of Participants by Group

Characteristic	No. (%)		*P* value
	Control (n = 107)	Insomnia (n = 53)	
Age, mean (SD), y	65.8 (4.4)	66.0 (5.0)	.85
Sex			
Female	53 (49.5)	31 (58.5)	.29
Male	54 (50.5)	22 (41.5)	
Race^a^			
African American/Black	9 (8.4)	10 (18.9)	.07
Asian	10 (9.3)	2 (3.8)	
Multiracial	5 (4.7)	0 (0.0)	
Pacific Islander	2 (1.9)	1 (1.9)	
White	81 (75.7)	40 (75.5)	
Ethnicity^a^			
Hispanic/Latino	7 (6.5)	4 (7.5)	.82
Non-Hispanic/Latino	100 (93.5)	49 (92.5)	
Marital status			
Married or partnered	47 (43.9)	24 (45.3)	.88
Income, mean (SD), $ in thousands	91.0 (56.9)	86.6 (51.8)	.66
Full-time employment	77 (72.0)	39 (73.6)	.83
Educational level, mean (SD), y	16.8 (2.3)	16.1 (1.5)	.02
BMI, mean (SD)^b^	24.9 (3.0)	25.2 (3.6)	.55
Charlson Comorbidity Index, mean (SD)^c^	2.2 (0.5)	2.4 (1.0)	.19
Sleep disturbance			
Duration of insomnia, mean (SD), y	NA	13.4 (11.4)	NA
Insomnia Severity Index, mean (SD)^d^	1.3 (1.8)	10.8 (5.3)	<.001
Use of hypnotic medications	0	1 (1.9)	.14
Depression			
History of depression^e^	14 (13.1)	15 (28.3)	.02
Use of antidepressants	0	0	.99
PHQ-8 score, mean (SD)^f^	0.3 (0.7)	2.7 (2.7)	<.001

Abbreviations: BMI, body mass index; NA, not applicable; PHQ-8, Patient Health Questionnaire 8 (PHQ-9 without insomnia item).

^a^ Race and ethnicity were self-reported by participants.

^b^ Calculated as weight in kilograms divided by height in meters squared.

^c^ The Charlson Comorbidity Index includes 17 categories of comorbidity, each with an assigned score of 1 to 6, depending on the risk of death associated with the condition; maximum score is 29.

^d^ Insomnia Severity Index rates severity of sleep disturbance according to *DSM-5* criteria. Scores range from 0 to 28, with a score <7 indicating no clinically significant insomnia.

^e^ Lifetime history of depression is fulfilled by major depressive disorder in the *DSM-5* criteria, as determined following administration of the Structured Clinical Interview.

^f^ PHQ-9 scores for each of the 9 criteria for major depressive disorder in the *DSM-IV-TR*, with 0 indicating not at all to 3 indicating nearly every day; maximum score of 27. All eligible insomnia participants had sleep disturbance; hence, PHQ-8 scored each of the criteria for major depressive disorder with the exception of insomnia, yielding a maximum score of 24. Scores <5 on the PHQ-9 indicate no to minimal depression.

### Adherence

Of those allocated, all 107 control participants and 53 insomnia participants completed the protocol. The number enrolled and in the intention-to-treat sample were identical.

### Inflammatory Response to Endotoxin

Endotoxin compared to placebo induced increases in IL-6 (condition effect, *F*_10,1496_ = 136.7; *P* < .001; eFigure 1A in Supplement 2), TNF (condition effect, *F*_10,1496_ = 396.1; *P* < .001; eFigure 1B in Supplement 2), and the composite of IL-6 and TNF (condition, *F*_10,1496_ = 276.8; *P* < .001, eFigure 1C in Supplement 2). No significant condition × group interactions were found, with similar effects adjusting for baseline.

### Primary Outcome

**Figure 2.**
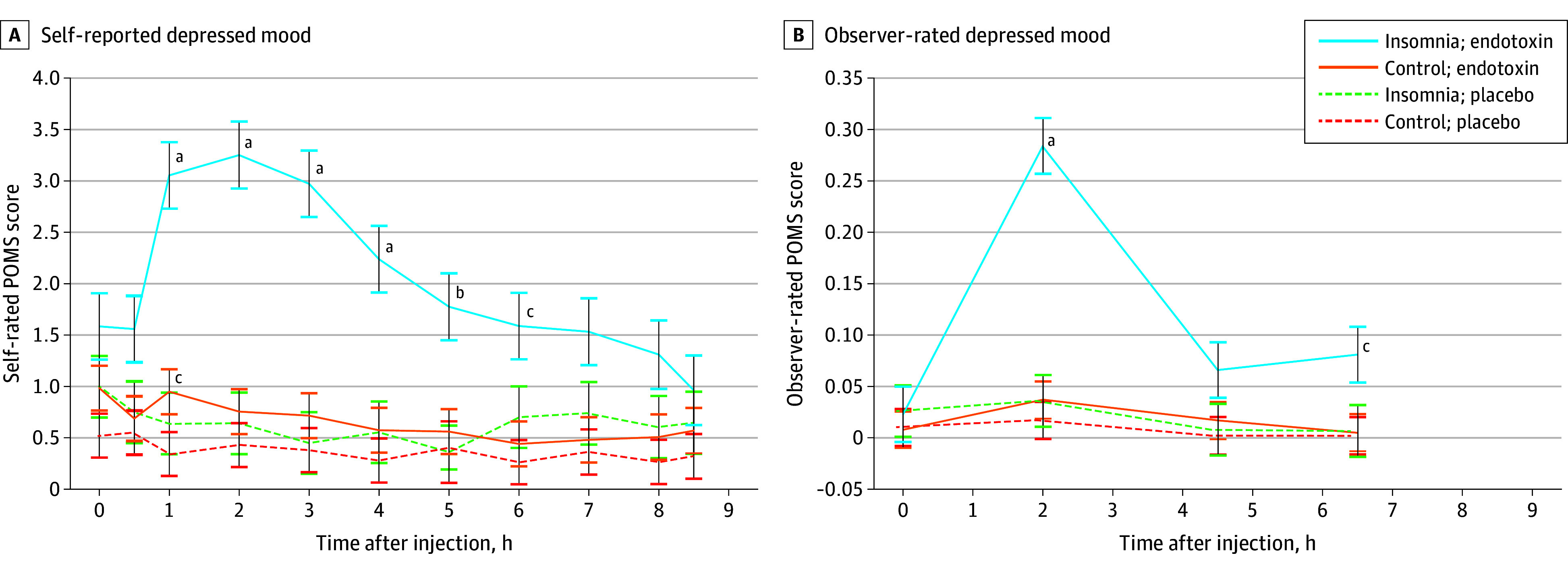
Changes in Depressed Mood (Primary Outcome) Changes over time in the endotoxin and placebo groups, stratified by insomnia status: self-reported (A) and observer-rated (B) depressed mood, as measured by the Profiles of Mood States depression subscale (POMS-D). Self-reported depressed mood was assessed at baseline, 30 minutes after infusion, and then hourly for 8 hours. Observer-rated depressed mood was assessed at baseline and then approximately every 2 hours. Time point condition differences (endotoxin vs placebo) were tested in the insomnia and control participants. Error bars depict the standard error of the mean. ^a^Significant differences at *P* < .001. ^b^Significant differences at *P* < .01. ^c^Significant differences at *P* < .05.

Endotoxin compared to placebo induced increases in the primary outcome, POMS-D (condition effect, *F*_10,1470_ = 7.3; *P* < .001), with greater responses in insomnia (condition × group, *F*_10,1470_ = 4.7; *P* < .001) and similar effects adjusting for baseline. Detailed model indices are reported in eTable 2 in Supplement 2. In insomnia, endotoxin vs placebo induced early and sustained increases in POMS-D (MD-AUC, 8.23; 95% CI, 1.50-14.96; T_1-4,_ all *P* values < .001; T_5_, *P* = .001; T_6_, *P* = .04), with no difference in control (MD-AUC, −1.65; 95% CI, −4.13 to 0.83; T_1_, *P* = .02; Figure 2A).

Endotoxin induced increases in observer-rated POMS-D (condition, *F*_3,450_ = 7.5; *P* < .001), with greater responses in insomnia (condition × group, *F*_3,450_ = 5.5; *P* = .001; Figure 2B) and similar effects adjusting for baseline. In insomnia, endotoxin vs placebo induced greater increases in observer-rated POMS-D (MD-AUC, 0.35; 95% CI, 0.03-0.67; T_2_, *P* < .001; T_6.5_, *P* = .01), with no difference in control (MD-AUC, 0.05; 95% CI, 0.00-0.09); no significant time point comparisons were found (Figure 2B).

**Figure 3.**
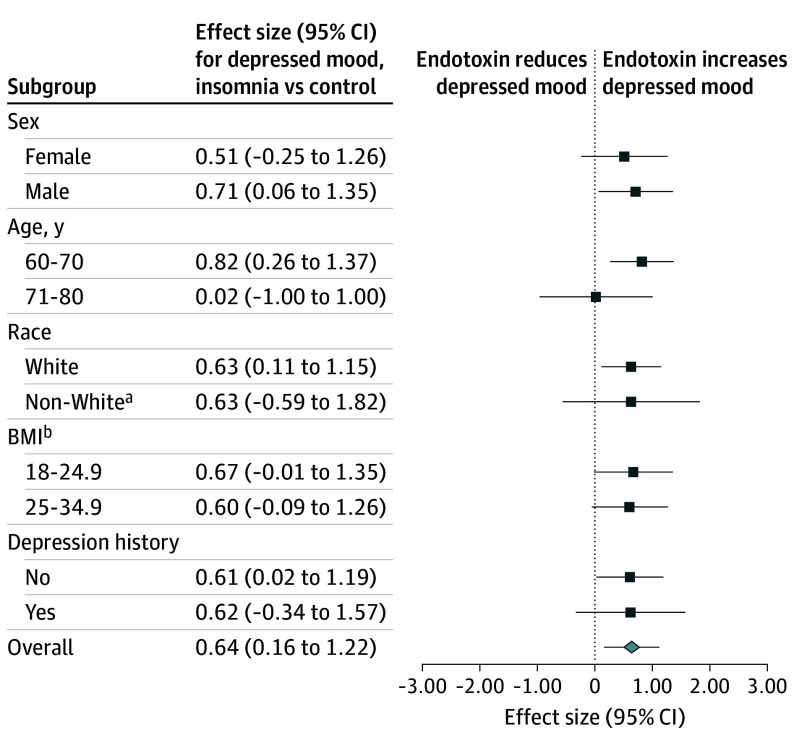
Insomnia Effect on Depressed Mood Within the Endotoxin Condition Effect size of differences in depressed mood between insomnia vs control participants in the endotoxin condition in the overall sample and subgroups. Race was self-reported by the participant. The no-effect point is 0 on the x-axis, as depicted by the dashed vertical line. The effect sizes for some subgroups had wide 95% confidence intervals owing to the small number of participants. BMI indicates body mass index. ^a^Non-White includes individuals reporting as African American/Black, Asian, multiracial, and Pacific Islander. Further testing of differences of non-White subgroup was not possible due to the small number in these subgroups. ^b^Calculated as weight in kilograms divided by height in meters squared.

Within the endotoxin condition, between-group (insomnia vs control) AUC effect sizes were calculated for POMS-D; insomnia showed greater increases in POMS-D (*d*, 0.64; 95% CI, 0.16-1.12) and observer-rated mood (*d*, 0.63; 95% CI, 0.15-1.11). Subgroups (age, 60-70 or 71-80 years; sex; race and ethnicity, White or non-White; BMI, 18-24.9 or 25-34.9) showed similar effects; effect was weaker in the oldest subgroup (Figure 3). Further testing of differences of non-White subgroup was not possible due to the small number in these subgroups.

Predefined sensitivity analyses examined the influence of sex. In female compared to male participants, endotoxin induced greater increases in POMS-D (condition × sex, *F*_10,1470_ = 1.7; *P* = .07) and observer-rated mood (condition × sex, *F*_3,453_ = 2.9; *P* = .04). No significant condition × group × sex interactions were found.

Additional sensitivity analyses examined time-varying influence of severe sickness symptoms on POMS-D. Endotoxin induced increases in severe sickness (condition, *F*_10,1485_ = 3.5; *P* < .001), with similar responses in groups (condition × group, *F*_10,1485_ = 1.0; *P* = .40). Severe sickness symptoms occurred in less than 15% of participants at any time point; effects were similar adjusting severe sickness symptoms for POMS-D (condition × group, *F*_10,1467_ = 5.0; *P* < .001) and observer-rated POMS-D (condition × group, *F*_3,448_ = 6.0; *P* < .001).

### Secondary Outcome: Observer-Rated Depressive Symptoms

**Figure 4.**
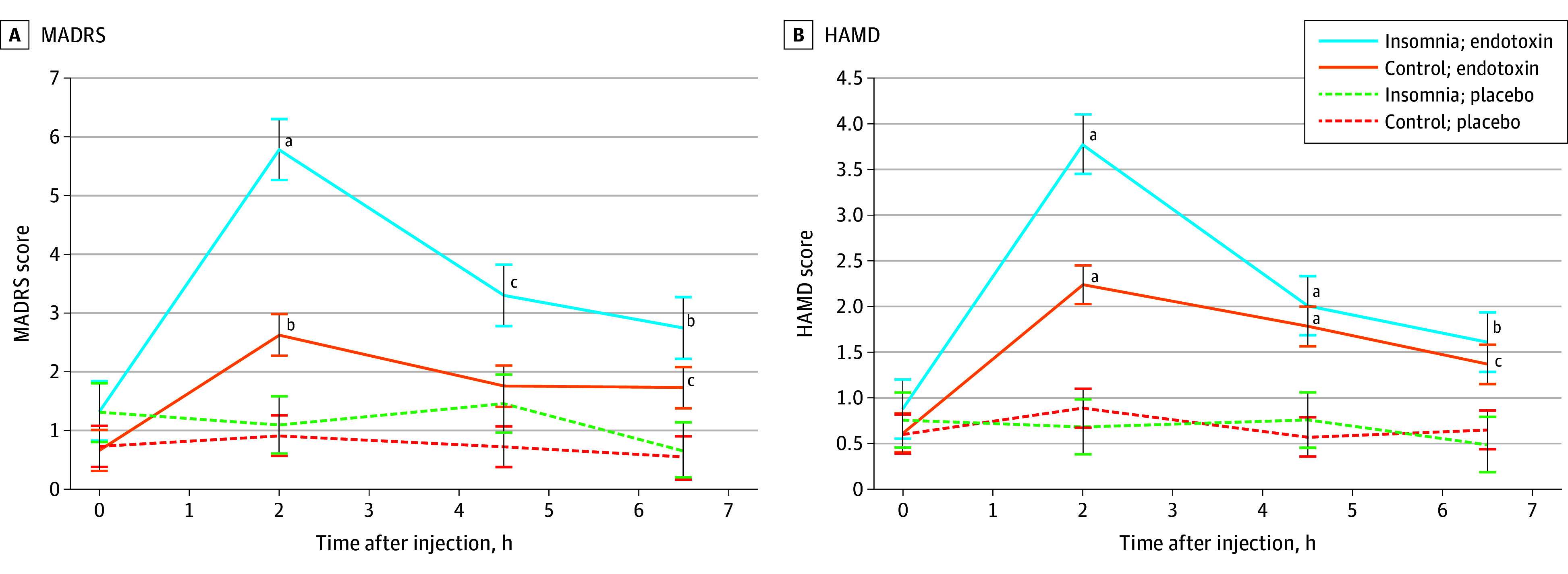
Changes in Depressive Symptoms (Secondary Outcomes) Changes over time in the endotoxin and placebo groups, stratified by insomnia status: severity of depressive symptoms as reported by the Montgomery-Åsberg Depression Rating Scale (MADRS) (A) and the Hamilton Rating Scale for Depression (HAMD) (B). Severity of depressive symptoms was assessed at baseline and then approximately every 2 hours. Time point condition differences (endotoxin vs placebo) were tested in the insomnia and control participants. Error bars depict the standard error of the mean. MADRS and HAMD scores are reported without scoring of insomnia items. ^a^Significant difference at *P* < .001. ^b^Significant difference at *P* < .01. ^c^Significant difference at *P* < .05.

Endotoxin induced increases in MADRS (condition, *F*_3,448_ = 13.8; *P* < .001), with greater responses in insomnia (condition × group, *F*_3,448_ = 2.9; *P* = .04; Figure 4A) and similar effects adjusting for baseline. In insomnia, endotoxin vs placebo induced a robust, clinically meaningful difference in MADRS,^51^ with differences at all time points (MD-AUC, 7.59; 95% CI, 1.47-13.70; T_2_, *P* < .001; T_4.5_, *P* = .02; T_6.5_, *P* = .006); no clinically meaningful increase was found in controls (MD-AUC, 3.98; 95% CI, 0.76-6.19; T_2_, *P* = .002; T_6.5_, *P* = .04).

Endotoxin induced increases in HAM-D (condition, *F*_3,450_ = 17.1; *P* < .001), with a greater response in insomnia (condition × group, *F*_3,450_ = 3.3; *P* = .02; Figure 4B) and similar effects adjusting for baseline (condition × group effect, *F*_3,450_ = 3.3; *P* = .03). In insomnia, endotoxin vs placebo induced a robust, clinically meaningful difference in HAM-D,^52^ with differences at all time points (MD-AUC, 4.38; 95% CI, 1.13-7.64; T_2,4.5_, *P* < .001; T_6.5_, *P* = .002); no clinically meaningful increase was found in controls (MD-AUC, 3.19; 95% CI, 1.74-4.64; T_2,4.5_, *P* < .001; T_6.5_, *P* = .02).

Sensitivity analyses within the insomnia group were performed to evaluate whether endotoxin induced increases in the MADRS and HAM-D within clinical thresholds.^53,54^ Both scales were rescored inclusive of insomnia items for comparative evaluation.^53,54^ In insomnia, endotoxin induced peak scores for MADRS (mean, 10.2; standard error of the mean [SEM], 7.8; 95% CI, 7.0-13.3) and HAM-D (mean, 7.2; SEM, 5.2; 95% CI, 5.0-9.32) in the “mild depression” range.^53,54^

Other secondary behavioral outcomes—social disconnection and anhedonia—showed similar results (eResults in Supplement 2). Adjustment for depression history did not alter any of the primary or secondary outcome results.

### Exploratory Analyses

Endotoxin induced robust increases in inflammatory and depressed mood responses. To explain the exaggerated increase in depressed mood in insomnia, 2 hypotheses were tested. First, it was hypothesized that endotoxin might induce an exaggerated increase in the inflammatory response in insomnia, which would biologically mediate exaggerated increases in POMS-D. However, inflammatory response was similar between groups. Further, a mediation analysis showed a direct effect of group and inflammatory composite on POMS-D (effect size, 9.10; 95% CI, 2.51-15.68; *P* = .007; effect size, 0.38; 95% CI, 0.14-0.63; *P* = .003). However the indirect effect was not significant (effect size, 0.19; 95% CI, −2.14 to 3.20; *P* = .35), indicating that exaggerated increases of POMS-D in insomnia were not mediated by differential increases in inflammation.

The second hypothesis tested whether participants with insomnia show an increased depression sensitivity to inflammatory exposure. As hypothesized, group interacted with the inflammatory composite to predict POMS-D (t(787.1) = 6.54; *P* < .001); within insomnia, the inflammatory composite correlated with POMS-D (β = 0.33; 95% CI, 0.26-0.41; *P* < .001) but not control (β = 0.02; 95% CI, −0.07 to 0.11; *P* = .61; eFigure 3 in Supplement 2), confirming the hypothesis that insomnia is associated with increased depression sensitivity to inflammatory exposure.

### Adverse Events

No adverse events occurred during the protocol. Phone interviews conducted 1 day and 7 days after the protocol found that MADRS scores were similar to baseline levels; no adverse physical events were reported.

## Discussion

In older adults with insomnia, inflammatory challenge induced exaggerated, 3-fold greater increases in depressed mood and depressive symptoms compared to those without insomnia. Moreover, among those with insomnia, depressed mood persisted for more than 6 hours, with only transient increases in older adults without insomnia. Older adults with insomnia showed a heightened depression sensitivity, as indexed by subjective and objective clinical measures, in response to inflammatory exposure, which was consistent across participant subgroups.

Most, if not all, studies examining depression responses to inflammatory challenge have enrolled healthy adults.^12,17,55^ Here, this first-of-its-kind experimental study in older adults supports mechanistic links between insomnia, inflammation, and depression^56,57^ and provides novel scientific evidence that older adults with insomnia are uniquely vulnerable to depressive symptoms following inflammatory exposure. Patients with insomnia might benefit from therapies that target inflammation-related depression.

A critical question is whether acute increases of depression responses to endotoxin are generalizable to development of diagnostic depression. Notably, this study assessed depressive symptoms using objective clinical scales. In insomnia, endotoxin induced a clinically important difference for MADRS and HAM-D,^51,52^ with peaks in the “mild depression” range for both MADRS and HAM-D; subthreshold depressive symptoms are known to predict diagnostic depression in older adults.^58^ Second, naturalistic inflammatory exposures (eg, infection, flare of an inflammatory disorder) yield greater and ongoing inflammation, possibly inducing persistent subthreshold depressive symptoms and depression development.^59,60^ Alternatively, exaggerated depression responses might have predictive validity, as affective state and hypothalamic-pituitary-adrenal axis response to initial IFN-a treatment predict later development of depression.^61,62^ Finally, exaggerated depression sensitivity indicates the need for active depression monitoring during times of inflammatory exposure, especially in older adults with insomnia^6^ with associated inflammaging and risk of infection.^4,5^

The mechanisms for depression sensitivity in insomnia are not known, although basic research has found that chronic sleep loss compromises the blood-brain barrier, resulting in migration of immune cells and inflammatory cytokines into the CNS and neuroinflammation (ie, microglial activation).^63,64^ In turn, neural pathways are primed to respond to acute inflammatory exposure; activated microglia convert kynurenine into a potent *N*-methyl-d-aspartate (NMDA) receptor agonist leading to increases in brain glutamate,^65^ notably in the dorsal anterior cingulate cortex implicated in endotoxin-induced depression.^42^ Alternatively, we have found that sleep loss alters the expression of clock genes in humans,^66^ and repeated sleep deprivation alters circadian oscillations of clock genes, which correlates with neuroinflammation and depressive behaviors in rats.^67^

It is thought that inflammatory activation contributes to depression.^11,68^ This study extends prior work and demonstrates that a behavioral phenotype (ie, insomnia) moderates risk of inflammation-related depression. In response to inflammatory challenge, the association between increases in inflammatory cytokines and depressed mood was significant for those with insomnia but not for those without insomnia. We also found that female participants show an increased depression sensitivity to endotoxin, consistent with prior observations,^16^ although female sex did not contribute to depression sensitivity to endotoxin in insomnia.

### Limitations

Chronic inflammation is likely to occur in older adults; hence, this study focused on older adults, and it is not known whether findings are generalizable to younger populations with insomnia. Nevertheless, a prior study showed that mild sleep disturbance was associated with depression sensitivity to endotoxin in young adult females,^43^ and subgroup analysis suggested greater depression sensitivity in those between 60 to 70 years old vs those older than 70 years, although this nonsignificant difference should be cautiously interpreted given the small number of participants older than 70 years. Second, because a disproportionate burden of risk for insomnia and depression is carried by those who are non-White,^60,69^ external validity in these subgroups requires further research. Third, the number of participants in the groups was imbalanced. Fourth, the experimental protocol examined the acute effect of endotoxin on depression responses. A longitudinal study is currently underway examining the predictive validity of depression sensitivity to endotoxin for the development of diagnostic depression.

## Conclusions

This rigorous, experimentally controlled randomized clinical trial found for the first time that inflammatory challenge with endotoxin induces increases in depressed mood and depressive symptoms in older adults and that depression responses were robustly exaggerated, both in magnitude and duration, in older adults with insomnia compared to those without insomnia. Experimental endotoxemia can be safely used to evaluate behavioral and biological mechanisms of affective risk in older adults. Strategies to monitor depression risk among older adults could be advanced by taking into account behavioral (ie, insomnia) and biologic (ie, inflammatory) phenotypes. Similarly, the efficacy of interventions to prevent late-life depression may be augmented by targeting both of these behavioral and biologic phenotypes.

## Notes

Supplement 1. Trial Protocol

Supplement 2. eMethods. Assessment of Inflammatory Markers eResults. Supplementary Results eTable 1. Characteristics of Participants, by Group and Conditiona eTable 2. Linear Mixed Model Indices for the Primary Outcome, POMS-D eFigure 1. Changes in Inflammatory Cytokines, Secondary Outcome eFigure 2. Changes in Feelings of Social Disconnection, Secondary Outcome eFigure 3. Changes in MADRS Anhedonia, Secondary Outcome eFigure 4. Association Between Depressed Mood and the Inflammatory Composite in the Insomnia Group and Controls eReference.

Supplement 3. Data Sharing Statement

## References

[1] Andreas S, Schulz H, Volkert J, . Prevalence of mental disorders in elderly people: the European MentDis_ICF65+ study. Br J Psychiatry. 2017;210(2):125-131. doi:10.1192/bjp.bp.115.180463 27609811

[2] Taylor WD. Clinical practice. depression in the elderly. N Engl J Med. 2014;371(13):1228-1236. doi:10.1056/NEJMcp1402180 25251617

[3] Cuijpers P, Vogelzangs N, Twisk J, Kleiboer A, Li J, Penninx BW. Differential mortality rates in major and subthreshold depression: meta-analysis of studies that measured both. Br J Psychiatry. 2013;202(1):22-27. doi:10.1192/bjp.bp.112.112169 23284149

[4] Furman D, Campisi J, Verdin E, . Chronic inflammation in the etiology of disease across the life span. Nat Med. 2019;25(12):1822-1832. doi:10.1038/s41591-019-0675-0 31806905 PMC7147972

[5] Franceschi C, Campisi J. Chronic inflammation (inflammaging) and its potential contribution to age-associated diseases. J Gerontol A Biol Sci Med Sci. 2014;69(suppl 1):S4-S9. doi:10.1093/gerona/glu057 24833586

[6] Piber D, Olmstead R, Cho JH, . Inflammaging: age and systemic, cellular, and nuclear inflammatory biology in older adults. J Gerontol A Biol Sci Med Sci. 2019;74(11):1716-1724. doi:10.1093/gerona/glz130 31107949 PMC6777092

[7] Cho HJ, Lavretsky H, Olmstead R, Levin MJ, Oxman MN, Irwin MR. Sleep disturbance and depression recurrence in community-dwelling older adults: a prospective study. Am J Psychiatry. 2008;165(12):1543-1550. doi:10.1176/appi.ajp.2008.07121882 18765482 PMC2707854

[8] Baglioni C, Battagliese G, Feige B, . Insomnia as a predictor of depression: a meta-analytic evaluation of longitudinal epidemiological studies. J Affect Disord. 2011;135(1-3):10-19. doi:10.1016/j.jad.2011.01.011 21300408

[9] Irwin MR, Carrillo C, Sadeghi N, Bjurstrom MF, Breen EC, Olmstead R. Prevention of incident and recurrent major depression in older adults with insomnia: a randomized clinical trial. JAMA Psychiatry. 2022;79(1):33-41. doi:10.1001/jamapsychiatry.2021.3422 34817561 PMC8733847

[10] Osimo EF, Pillinger T, Rodriguez IM, Khandaker GM, Pariante CM, Howes OD. Inflammatory markers in depression: a meta-analysis of mean differences and variability in 5,166 patients and 5,083 controls. Brain Behav Immun. 2020;87:901-909. doi:10.1016/j.bbi.2020.02.010 32113908 PMC7327519

[11] Miller AH, Raison CL. The role of inflammation in depression: from evolutionary imperative to modern treatment target. Nat Rev Immunol. 2016;16(1):22-34. doi:10.1038/nri.2015.5 26711676 PMC5542678

[12] Lasselin J, Lekander M, Benson S, Schedlowski M, Engler H. Sick for science: experimental endotoxemia as a translational tool to develop and test new therapies for inflammation-associated depression. Mol Psychiatry. 2021;26(8):3672-3683. doi:10.1038/s41380-020-00869-2 32873895 PMC8550942

[13] Suffredini AF, Noveck RJ. Human endotoxin administration as an experimental model in drug development. Clin Pharmacol Ther. 2014;96(4):418-422. doi:10.1038/clpt.2014.146 25236665

[14] Hannestad J, Gallezot JD, Schafbauer T, . Endotoxin-induced systemic inflammation activates microglia: [^11^C]PBR28 positron emission tomography in nonhuman primates. Neuroimage. 2012;63(1):232-239. doi:10.1016/j.neuroimage.2012.06.055 22776451 PMC3699786

[15] Sandiego CM, Gallezot JD, Pittman B, . Imaging robust microglial activation after lipopolysaccharide administration in humans with PET. Proc Natl Acad Sci U S A. 2015;112(40):12468-12473. doi:10.1073/pnas.1511003112 26385967 PMC4603509

[16] Moieni M, Irwin MR, Jevtic I, Olmstead R, Breen EC, Eisenberger NI. Sex differences in depressive and socioemotional responses to an inflammatory challenge: implications for sex differences in depression. Neuropsychopharmacology. 2015;40(7):1709-1716. doi:10.1038/npp.2015.17 25598426 PMC4915253

[17] Schedlowski M, Engler H, Grigoleit JS. Endotoxin-induced experimental systemic inflammation in humans: a model to disentangle immune-to-brain communication. Brain Behav Immun. 2014;35:1-8. doi:10.1016/j.bbi.2013.09.015 24491305

[18] Irwin MR, Boyle CC, Cho JH, . Sleep and Healthy Aging Research on Depression (SHARE-D) randomized controlled trial: protocol overview of an experimental model of depression with insomnia, inflammation, and affect mechanisms in older adults. Brain Behav Immun Health. 2023;28:100601. doi:10.1016/j.bbih.2023.100601 36879913 PMC9984307

[19] Runyan CW, Johnson RM, Yang J, . Risk and protective factors for fires, burns, and carbon monoxide poisoning in U.S. households. Am J Prev Med. 2005;28(1):102-108. doi:10.1016/j.amepre.2004.09.014 15626564 PMC3066116

[20] O’Malley AS, Forrest CB. Beyond the examination room: primary care performance and the patient-physician relationship for low-income women. J Gen Intern Med. 2002;17(1):66-74. doi:10.1046/j.1525-1497.2002.10338.x 11903777 PMC1495002

[21] Irwin MR, Cole S, Olmstead R, . Moderators for depressed mood and systemic and transcriptional inflammatory responses: a randomized controlled trial of endotoxin. Neuropsychopharmacology. 2019;44(3):635-641. doi:10.1038/s41386-018-0259-6 30391995 PMC6333799

[22] Irwin MR, Olmstead R, Breen EC, . Cognitive behavioral therapy and tai chi reverse cellular and genomic markers of inflammation in late-life insomnia: a randomized controlled trial. Biol Psychiatry. 2015;78(10):721-729. doi:10.1016/j.biopsych.2015.01.010 25748580 PMC4524803

[23] Black DS, O’Reilly GA, Olmstead R, Breen EC, Irwin MR. Mindfulness meditation and improvement in sleep quality and daytime impairment among older adults with sleep disturbances: a randomized clinical trial. JAMA Intern Med. 2015;175(4):494-501. doi:10.1001/jamainternmed.2014.8081 25686304 PMC4407465

[24] Irwin MR, Olmstead R, Carrillo C, . Cognitive behavioral therapy vs. tai chi for late life insomnia and inflammatory risk: a randomized controlled comparative efficacy trial. Sleep. 2014;37(9):1543-1552. doi:10.5665/sleep.4008 25142571 PMC4153053

[25] Drill R, Nakash O, DeFife JA, Westen D. Assessment of clinical information: comparison of the validity of a Structured Clinical Interview (the SCID) and the Clinical Diagnostic Interview. J Nerv Ment Dis. 2015;203(6):459-462. doi:10.1097/NMD.0000000000000300 25974055 PMC4452387

[26] First MB, Spitzer RL, Gibbon M, Williams JB. Structured Clinical Interview for DSM-IV Axis I Disorders - Patient Edition, Version 2.0. New York State Psychiatric Institute; 1996.

[27] Scott NW, McPherson GC, Ramsay CR, Campbell MK. The method of minimization for allocation to clinical trials. a review. Control Clin Trials. 2002;23(6):662-674. doi:10.1016/S0197-2456(02)00242-8 12505244

[28] Moieni M, Tan KM, Inagaki TK, . Sex differences in the relationship between inflammation and reward sensitivity: a randomized controlled trial of endotoxin. Biol Psychiatry Cogn Neurosci Neuroimaging. 2019;4(7):619-626. doi:10.1016/j.bpsc.2019.03.010 31103547 PMC6612452

[29] Suffredini AF, Hochstein HD, McMahon FG. Dose-related inflammatory effects of intravenous endotoxin in humans: evaluation of a new clinical lot of Escherichia coli O:113 endotoxin. J Infect Dis. 1999;179(5):1278-1282. doi:10.1086/314717 10191237

[30] Musselman DL, Lawson DH, Gumnick JF, . Paroxetine for the prevention of depression induced by high-dose interferon alfa. N Engl J Med. 2001;344(13):961-966. doi:10.1056/NEJM200103293441303 11274622

[31] Harrison NA, Brydon L, Walker C, Gray MA, Steptoe A, Critchley HD. Inflammation causes mood changes through alterations in subgenual cingulate activity and mesolimbic connectivity. Biol Psychiatry. 2009;66(5):407-414. doi:10.1016/j.biopsych.2009.03.015 19423079 PMC2885494

[32] Balter LJT, Hulsken S, Aldred S, . Low-grade inflammation decreases emotion recognition - evidence from the vaccination model of inflammation. Brain Behav Immun. 2018;73:216-221. doi:10.1016/j.bbi.2018.05.006 29742460

[33] Shacham S. A shortened version of the Profile of Mood States. J Pers Assess. 1983;47(3):305-306. doi:10.1207/s15327752jpa4703_14 6886962

[34] Norcross JC, Guadagnoli E, Prochaska JO. Factor structure of the Profile of Mood States (POMS): two partial replications. J Clin Psychol. 1984;40(5):1270-1277. doi:10.1002/1097-4679(198409)40:5<1270::AID-JCLP2270400526>3.0.CO;2-7 6490926

[35] McNair DM, Lorr M, Droppleman LF. Manual for the Profile of Mood States. Educational and Industrial Testing Service; 1992.

[36] Hammond MF. Rating depression severity in the elderly physically ill patient: reliability and factor structure of the Hamilton and the Montgomery-Asberg Depression Rating Scales. Int J Geriatr Psychiatry. 1998;13(4):257-261. doi:10.1002/(SICI)1099-1166(199804)13:4<257::AID-GPS773>3.0.CO;2-U 9646154

[37] Brown C, Schulberg HC, Madonia MJ. Assessing depression in primary care practice with the Beck Depression Inventory and the Hamilton Rating Scale for Depression. Psychol Assess. 1995;7:59-65. doi:10.1037/1040-3590.7.1.59

[38] Endicott J, Cohen J, Nee J, Fleiss J, Sarantakos S. Hamilton Depression Rating Scale. extracted from regular and change versions of the Schedule for Affective Disorders and Schizophrenia. Arch Gen Psychiatry. 1981;38(1):98-103. doi:10.1001/archpsyc.1981.01780260100011 7458574

[39] Heinrich LM, Gullone E. The clinical significance of loneliness: a literature review. Clin Psychol Rev. 2006;26(6):695-718. doi:10.1016/j.cpr.2006.04.002 16952717

[40] Piber D, Olmstead R, Cho JH, Guzman M, Irwin MR. Interferon-*γ* moderation of poor sleep maintenance and depressed mood in community-dwelling older adults. Psychol Med. 2023;53(8):3548-3556. doi:10.1017/S0033291722000113 35144705

[41] Eisenberger NI, Inagaki TK, Mashal NM, Irwin MR. Inflammation and social experience: an inflammatory challenge induces feelings of social disconnection in addition to depressed mood. Brain Behav Immun. 2010;24(4):558-563. doi:10.1016/j.bbi.2009.12.009 20043983 PMC2856755

[42] Eisenberger NI, Inagaki TK, Rameson LT, Mashal NM, Irwin MR. An fMRI study of cytokine-induced depressed mood and social pain: the role of sex differences. Neuroimage. 2009;47(3):881-890. doi:10.1016/j.neuroimage.2009.04.040 19376240 PMC2733873

[43] Cho HJ, Eisenberger NI, Olmstead R, Breen EC, Irwin MR. Preexisting mild sleep disturbance as a vulnerability factor for inflammation-induced depressed mood: a human experimental study. Transl Psychiatry. 2016;6(3):e750. doi:10.1038/tp.2016.23 26954978 PMC4872448

[44] Turner L, Shamseer L, Altman DG, . Consolidated standards of reporting trials (CONSORT) and the completeness of reporting of randomised controlled trials (RCTs) published in medical journals. Cochrane Database Syst Rev. 2012;11(11):MR000030. doi:10.1002/14651858.MR000030.pub2 23152285 PMC7386818

[45] O’Connor MF, Irwin MR. Links between behavioral factors and inflammation. Clin Pharmacol Ther. 2010;87(4):479-482. doi:10.1038/clpt.2009.255 20130566 PMC2866374

[46] O’Connor MF, Bower JE, Cho HJ, . To assess, to control, to exclude: effects of biobehavioral factors on circulating inflammatory markers. Brain Behav Immun. 2009;23(7):887-897. doi:10.1016/j.bbi.2009.04.005 19389469 PMC2749909

[47] Hayes AF. An index and test of linear moderated mediation. Multivariate Behav Res. 2015;50(1):1-22. doi:10.1080/00273171.2014.962683 26609740

[48] Hayes AF, Preacher KJ. Quantifying and testing indirect effects in simple mediation models when the constituent paths are nonlinear. Multivariate Behav Res. 2010;45(4):627-660. doi:10.1080/00273171.2010.498290 26735713

[49] Preacher KJ, Hayes AF. SPSS and SAS procedures for estimating indirect effects in simple mediation models. Behav Res Methods Instrum Comput. 2004;36(4):717-731. doi:10.3758/BF03206553 15641418

[50] Hofmann SG, Curtiss JE, Hayes SC. Beyond linear mediation: toward a dynamic network approach to study treatment processes. Clin Psychol Rev. 2020;76:101824. doi:10.1016/j.cpr.2020.101824 32035297 PMC7137783

[51] Turkoz I, Alphs L, Singh J, . Clinically meaningful changes on depressive symptom measures and patient-reported outcomes in patients with treatment-resistant depression. Acta Psychiatr Scand. 2021;143(3):253-263. doi:10.1111/acps.13260 33249552 PMC7986932

[52] Rush AJ, South C, Jain S, . Clinically significant changes in the 17- and 6-Item Hamilton Rating Scales for Depression: a STAR*D report. Neuropsychiatr Dis Treat. 2021;17:2333-2345. doi:10.2147/NDT.S305331 34295161 PMC8290193

[53] Thase ME, Harrington A, Calabrese J, Montgomery S, Niu X, Patel MD. Evaluation of MADRS severity thresholds in patients with bipolar depression. J Affect Disord. 2021;286:58-63. doi:10.1016/j.jad.2021.02.043 33677183

[54] Zimmerman M, Martinez JH, Young D, Chelminski I, Dalrymple K. Severity classification on the Hamilton Depression Rating Scale. J Affect Disord. 2013;150(2):384-388. doi:10.1016/j.jad.2013.04.028 23759278

[55] DellaGioia N, Hannestad J. A critical review of human endotoxin administration as an experimental paradigm of depression. Neurosci Biobehav Rev. 2010;34(1):130-143. doi:10.1016/j.neubiorev.2009.07.014 19666048 PMC2795398

[56] Irwin MR. Why sleep is important for health: a psychoneuroimmunology perspective. Annu Rev Psychol. 2015;66:143-172. doi:10.1146/annurev-psych-010213-115205 25061767 PMC4961463

[57] Irwin MR, Piber D. Insomnia and inflammation: a two hit model of depression risk and prevention. World Psychiatry. 2018;17(3):359-361. doi:10.1002/wps.20556 30229570 PMC6127743

[58] Karsten J, Hartman CA, Smit JH, . Psychiatric history and subthreshold symptoms as predictors of the occurrence of depressive or anxiety disorder within 2 years. Br J Psychiatry. 2011;198(3):206-212. doi:10.1192/bjp.bp.110.080572 21357879

[59] Irwin MR, Straub RH, Smith MT. Heat of the night: sleep disturbance activates inflammatory mechanisms and induces pain in rheumatoid arthritis. Nat Rev Rheumatol. 2023;19(9):545-559. doi:10.1038/s41584-023-00997-3 37488298

[60] Slavich GM, Irwin MR. From stress to inflammation and major depressive disorder: a social signal transduction theory of depression. Psychol Bull. 2014;140(3):774-815. doi:10.1037/a0035302 24417575 PMC4006295

[61] Capuron L, Ravaud A. Prediction of the depressive effects of interferon alfa therapy by the patient’s initial affective state. N Engl J Med. 1999;340(17):1370. doi:10.1056/NEJM199904293401716 10223879

[62] Capuron L, Raison CL, Musselman DL, Lawson DH, Nemeroff CB, Miller AH. Association of exaggerated HPA axis response to the initial injection of interferon-alpha with development of depression during interferon-alpha therapy. Am J Psychiatry. 2003;160(7):1342-1345. doi:10.1176/appi.ajp.160.7.1342 12832253

[63] Herrero Babiloni A, Baril AA, Charlebois-Plante C, . The putative role of neuroinflammation in the interaction between traumatic brain injuries, sleep, pain and other neuropsychiatric outcomes: a state-of-the-art review. J Clin Med. 2023;12(5):1793. doi:10.3390/jcm12051793 36902580 PMC10002551

[64] Hurtado-Alvarado G, Domínguez-Salazar E, Pavon L, Velázquez-Moctezuma J, Gómez-González B. Blood-brain barrier disruption induced by chronic sleep loss: low-grade inflammation may be the link. J Immunol Res. 2016;2016:4576012. doi:10.1155/2016/4576012 27738642 PMC5050358

[65] Haroon E, Miller AH. Rewiring the brain: inflammation’s impact on glutamate and neural networks in depression. Neuropsychopharmacology. 2024;50(1):312-313. doi:10.1038/s41386-024-01921-3 39020141 PMC11525645

[66] Irwin MR, Wang M, Campomayor CO, Collado-Hidalgo A, Cole S. Sleep deprivation and activation of morning levels of cellular and genomic markers of inflammation. Arch Intern Med. 2006;166(16):1756-1762. doi:10.1001/archinte.166.16.1756 16983055

[67] Xing C, Zhou Y, Xu H, . Sleep disturbance induces depressive behaviors and neuroinflammation by altering the circadian oscillations of clock genes in rats. Neurosci Res. 2021;171:124-132. doi:10.1016/j.neures.2021.03.006 33785408

[68] Haroon E, Fleischer CC, Felger JC, . Conceptual convergence: increased inflammation is associated with increased basal ganglia glutamate in patients with major depression. Mol Psychiatry. 2016;21(10):1351-1357. doi:10.1038/mp.2015.206 26754953 PMC4940313

[69] Grandner MA, Williams NJ, Knutson KL, Roberts D, Jean-Louis G. Sleep disparity, race/ethnicity, and socioeconomic position. Sleep Med. 2016;18:7-18. doi:10.1016/j.sleep.2015.01.020 26431755 PMC4631795

