# Supplementary material for: Inflammatory Exposure and Depression in Older Adults With Insomnia: A Randomized Clinical Trial
Source: JAMA Psychiatry. 2025 Jul 16;82(9):859–67. doi: 10.1001/jamapsychiatry.2025.1327 (PMC12268530; doi:10.1001/jamapsychiatry.2025.1327)
Supplement: Supplement 1. — Trial Protocol [file jamapsychiatry-e251327-s001.pdf]

Sleep and Healthy Aging Research on Depression (SHARE-D) Randomized Controlled Trial: Protocol of an Experimental Model of Depression with Insomnia, Inflammation, and Affect Mechanisms in Older Adults

Overview of Study

Depression, one of the most common diseases in older adults, carries significant risk for morbidity and mortality. Because of the burgeoning population of older adults, the enormous burden of late-life depression, and the limited efficacy of current antidepressants in older adults, biologically plausible models that translate into selective depression prevention strategies are needed. Insomnia predicts depression recurrence and is a modifiable target to prevent incident and recurrent depression in older adults. Yet, it is not known how insomnia gets converted into biological- and affective risk for depression, which is critical for identification of molecular targets for pharmacologic interventions, and for refinement of insomnia treatments that target affective responding to improve efficacy. Sleep disturbance activates inflammatory signaling and primes immune responses to subsequent inflammatory challenge. In turn, inflammatory challenge induces depressive symptoms, which correlate with activation of brain regions implicated in depression. This study hypothesizes that insomnia serves as a vulnerability factor for inflammation-related depression; older adults with insomnia will show heightened inflammatory- and affective responding to inflammatory challenge as compared to those without insomnia. To test this hypothesis, this protocol paper describes a placebo-controlled, randomized, double-blind study of low dose endotoxin in older adults (n=160; 60-80 y; stratified by sex) with insomnia vs. comparison controls without insomnia. The aims of this study are to examine differences in depressive symptoms, measures of negative affective responding, and measures of positive affective responding as a function of insomnia and inflammatory challenge. If the

hypotheses are confirmed, older adults with two “hits”, insomnia and inflammatory activation, would represent a high risk group to be prioritized for monitoring and for depression prevention efforts using treatments that target insomnia or inflammation. Moreover, this study will inform the development of mechanism-based treatments that target affect responses in addition to sleep behaviors, and which might also be coupled with efforts to reduce inflammation to optimize efficacy of depression prevention.

Background

Depression, one of the most common diseases in older adults, carries significant risk for morbidity and mortality [1,2]. Many older adults with depression are not identified [3], and even when identified, over 60% of older adults fail to achieve symptomatic remission [4,5]. Because of the burgeoning population of older adults, the enormous burden of late-life depression, and the limited efficacy of current antidepressants in older adults, biologically plausible models that can be translated into depression prevention efforts are needed.

Insomnia predicts depression recurrence [6] especially in older adults [7,8] and is a modifiable target for depression prevention. Indeed, we have found that treatment of insomnia prevents incident and recurrent major depressive disorder in non-depressed older adults [9]. Yet, it is not known how insomnia gets converted into biological- and affective risk for depression, which is critical for identification of molecular targets for pharmacologic interventions, and for refinement of insomnia treatments that target affective responding with the potential to improve efficacy.

Sleep disturbance and/or insomnia activate inflammatory signaling and prime immune responses to subsequent inflammatory challenge [10]. In addition, substantial evidence links inflammation to depression, as reviewed previously [11,12]. Further, we and others have found that inflammation has a causative role in inducing depressive symptoms [12]. For example, acute inflammatory challenge (i.e., endotoxin) induces depressive symptoms [13-16], which correlate with activation of brain regions implicated in depression [16-18]. Finally, sleep disturbance is reported to be associated with exaggerated increases in depressive symptoms in response to endotoxin [19],

with greater increases in women vs. men [19]. Given this evidence, the present study hypothesizes that insomnia serves as a vulnerability factor for inflammation-related depression [20]. We hypothesize that older adults with insomnia will show heightened affective responding to inflammatory challenge as compared to those without insomnia.

It is important to study older adults with insomnia, as “usual” aging is associated with inflammation [21-23]. In addition, insomnia is highly prevalent in older adults and can exacerbate age-related inflammation [10,24]. Together, insomnia and pre-existing inflammation, might heighten affective responding to inflammatory challenge. Whereas some prospective evidence suggests that chronic inflammation predicts depressive symptoms [25,26], findings are mixed [27,28]. Nevertheless, experimental studies have found that chronic inflammation primes inflammatory activation [23,29] and heightens depressive symptoms in response to inflammatory challenge [12,30-33], including challenges due to biologic (i.e. infections) and psychosocial factors (i.e., interpersonal stress) [12,34,35]. Moreover, inflammatory reactivity predicts acute increases in depressive symptoms [12,36] as well as increases in depressive symptoms over the following year [36]. Conversely in depressed patients, acute decreases in inflammation are associated with clinically meaningful decreases in depressive symptoms (e.g., ketamine treatment) [37-39].

Depression in Older Adults: Need for Prevention Approaches

Depression in older adults is a major public health concern. Given that older adults with depression often do not receive diagnosis and treatment [3], and only about 30-35% of older adults achieve remission using current treatment approaches [40] with over two-thirds of the disease burden remaining [41,42], innovative approaches that selectively prevent depression are needed [43]. Furthermore, the Institutes of Medicine has long called for efforts to develop, evaluate, and implement interventions focused on depression prevention [44,45]. However, for prevention strategies to be efficient, it is necessary to target subgroups at high risk (i.e.,

selective prevention) [46], as we have recently demonstrated in a selective prevention trial, in which treatment of insomnia prevented incident and recurrent major depressive disorder in older adults with insomnia [9]. However, the mechanisms that contribute to the efficacy of insomnia treatment to prevent depression are not known. The present study is significant by being the first to use an experimental approach to evaluate the independent and interactive effects of two modifiable risk factors, insomnia and inflammation, on depressive symptoms and mechanisms of negative and positive affective responding, and to do so in a vulnerable population of older adults.

Prevention of Depression in Older Adults: Significance of Insomnia

To maximize efficiency of a prevention intervention in older adults, it is important to identify modifiable risk factors that can be targeted [43,47,48]. Insomnia is associated with a nearly 2-fold increase in depression risk [6,49], with further elevated risk in older adults [7,8]. Treatment of insomnia with cognitive behavioral therapy for insomnia (CBT-I), as compared to sleep education therapy, reduces incident and recurrent major depressive disorder by 51% in older adults with insomnia [9]. The proposed study is significant by experimentally examining whether the effects of insomnia on depression risk is differentially triggered by inflammation. Findings will provide understanding to inform the refinement of prevention efforts at three levels of analysis: population selection (i.e., targeting those with insomnia and/or inflammatory disorder), timing of intervention (i.e., delivery of the intervention during onset of inflammatory disorder and/or infection), and target of intervention (i.e., sleep, affective responses).

Insomnia as a Modifiable Risk Factor: Need for Treatment Optimization

Insomnia is associated with difficulties in down-regulating negative affect, with increased reactivity to negative affective stimuli as measured by psychophysiological responses [50,51]. Less is known about the effects of insomnia on positive affect systems, although insomnia appears to

be associated with difficulty up-regulating positive affect (i.e., anhedonia) [52]. Difficulties with affective responding might also have reciprocal effects on sleep and perpetuate insomnia by activating arousal mechanisms [53-55]. Behavioral treatments such as cognitive behavioral therapy for insomnia (CBT-I) target sleep behaviors, but the efficacy of CBT-I is often no better than 50% [56]. The proposed study is significant by providing experimental insight into the impact of insomnia on affective responding, which can inform the development of adjunctive insomnia treatment components that target specific processes related to affect responding (i.e., social rejection sensitivity, negative bias in facial emotion recognition) or reward deficits, the latter of which could inform the development of interventions that pharmacologically target neurotransmitters (i.e., dopamine) implicated in reward.

Inflammation-Induced Depression: An Experimental Model in Older Adults

Given that sleep disturbance leads to daytime increases in circulating levels of inflammatory cytokines and C-reactive protein (CRP) [10,57-60], that modest amounts of sleep loss activate cellular and inflammatory nuclear signaling pathways (e.g., nuclear factor [NF]-κB) [61,62], and that treatment of insomnia promotes a reversal of systemic and cellular inflammation in older adults [63,64], abundant evidence supports the link between insomnia inflammatory activation. Further, “usual” aging is also associated with elevations in markers of inflammation [29], which leads to “inflammaging” or low-grade, chronic, systemic inflammation [21,23]. However, even in those with chronic increases in inflammation, there is dynamic variability, due to in part to many other contributing factors that acutely increase inflammation including specific diseases (i.e. infections) [34,35] and psychosocial factors (i.e., interpersonal stress) [12]. Indeed, when acute increases in inflammation occur, especially in those with chronic inflammation in which the immune system is primed, the likelihood of depression may be heightened [12,65,66]. For example, acute inflammatory reactivity to laboratory-based tasks of interpersonal threat correlates with increases in depressed mood [12], and increases in inflammation and depressive symptoms are

both exaggerated in those in those with inflammatory conditions [30,67]. Importantly, such acute increases in inflammatory markers are clinically meaningful, as greater inflammatory reactivity predicts increases in depression over the following year [12,36], and conversely acute decreases in proinflammatory cytokines are associated with clinically meaningful decreases in depressive symptoms following treatment with ketamine [37,68,69].

Endotoxin is a model of systemic inflammation that can be used to experimentally interrogate the role of inflammation to induce depressive symptoms [13]. Endotoxin resembles a pathogen-induced, naturally occurring inflammatory immune response, which is driven by a complex interplay of various cytokines, all with distinct kinetics and locally differing concentrations [14,70]. Injection of a single cytokine does not model this response as it does not target the inflammatory cascade. Likewise, acute laboratory-based stress can activate inflammation, but only induces modest inflammatory and depression responses, which are less robust than found with endotoxin.

Increasingly, depression is viewed along a continuum of affective responses that lead to symptom expression, as opposed to being a categorical, diagnostic construct [71,72], and endotoxin affects the two cardinal affective symptoms that constitute depression: depressed mood and anhedonia. Endotoxin induces increases in depressed mood (Profile of Mood States [POMS]>3), and these responses are nearly 2-fold greater and within the range of clinical severity in those with sleep disturbance [19]. Similarly, endotoxin induces increases depressive symptoms as indexed by the Montgomery-Asberg Depression Rating Scale (MADRS > 7), indicating mild depression [73], and these responses are attenuated by pretreatment with the antidepressant citalopram [73]. Anhedonia (i.e., the lack of pleasure) also increases in response to endotoxin [18,73]. Finally, endotoxin administration induces acute changes in the activity of neural substrates that are linked to depression. For example, the dorsal anterior cingulate cortex (dACC) and its functional connectivity to other regions are recognized for their role in the pathophysiology of depression. Endotoxin induces increases in activity of the dACC, which are

correlated with increases in inflammatory cytokines and reports of depressed mood [16]. Moreover, endotoxin administration also reduces neural activity in the ventral striatum, a reward-related neural region [18], and decreased ventral striatum activity mediates effects of endotoxin on increases in depressed mood [18].

No prior research has used the endotoxin model, or any other inflammatory challenge, to probe depression risk in older adults. Moreover, no prior study has tested the role of insomnia in the moderation of depression responses, nor used objective assessment of negative- and positive affective responding to inform the development of treatments that target these affective pathways. This study will address each of the limitations of prior research by evaluating older adults and by examining depression responses as a function of insomnia with objective assessment of depressive symptoms and task based affective responding.

In this placebo-controlled, randomized, double-blind study of acute systemic inflammation in older adults (aged 60 to 80 years; stratified by sex) with insomnia vs. comparison controls without insomnia, we aim to examine differences in measures of depressive symptoms (primary outcome) and measures of negative affective responding (secondary outcome) in response to inflammatory challenge with low-dose endotoxin as a function of insomnia. Additionally, we aim to examine differences in measures of positive affective responding (secondary outcome). A further aim of this study is to evaluate whether differences in depressive symptoms, negative affective responding, and positive affective responding correlate with increases in inflammation in response to endotoxin as a function of insomnia. As an exploratory aim, this study will examine whether sex differences explain variability in depressive symptoms, negative affective responding, and positive affective responding in response to inflammatory challenge as a function of insomnia, given prior results in adults [15].

METHODS

Trial Design

This investigation is a randomized controlled trial to evaluate the effects of an acute inflammatory challenge in response to endotoxin (0.8 ng/kg body weight) vs. placebo over 12 hours in older adults with insomnia as compared to older adults without insomnia. One hundred sixty older adults (aged 60 to 80 years), who are free from current psychiatric illness and medical conditions (e.g., inflammatory disorders), will be entered into this placebo-controlled, randomized, double-blind, parallel arm (endotoxin vs. placebo) experimental challenge protocol. Of this sample, approximately 60 participants will fulfill DSM-5 criteria for an insomnia disorder. Given the feasibility and cost utility of recruiting comparison controls who do not have insomnia, 100 controls will be used. Furthermore, a larger group of comparison controls lends more statistical power for the overall study, as well as greater statistical power for secondary analyses related, for example, to sex differences in depressive and inflammatory responses in older adults. Within each group, randomized conditions of endotoxin vs. placebo will be balanced with a 1:1 randomization ratio. Three pre-entry classification variables will be used to balance participants between those with insomnia and comparison controls including age (age 60-70 vs. 71-80), sex, and body mass index (18-24.9 vs. 25–35 kg/m^2). We will also use these pre-treatment classification variables in a modified randomization procedure, the minimization method [74] to ensure that the endotoxin vs. placebo conditions in the study design are balanced. The minimization method is particularly useful when it is important to balance experimental groups on a larger number of covariates [74]. In its most simple application, this method weighs each classification variable equally and seeks to achieve an overall balance of the levels of these variables across experimental conditions, rather than a balance within each stratum. In addition, we will measure possible moderators (i.e., severity/ duration of insomnia, prior depressive episodes) and examine their potential moderating roles.

Participants will provide written informed consent as approved by the UCLA (University of California, Los Angeles) Institutional Review Board, as further described in the section on

Ethics. All data were deidentified. Trial data monitoring and steer-ing committees of the UCLA Clinical Translational Sciences Institute oversaw the study, which was undertaken according to the intention-to-treat principle. This study will adhere to the Consolidated Standards of Reporting Trials (CONSORT) reporting guideline.

Participants will be randomly assigned to receive either endotoxin or placebo in a 1:1 ratio in a between-subjects manner, stratified by insomnia status. Following administration of endotoxin (0.8 ng/kg body weight) vs. placebo, self- and observer rated measures of depressed mood will be assessed hourly over 12 hours (primary outcome) and self-rated measures of depressed mood and depressive symptoms every two hours over 12 hours (primary outcome). Additional secondary outcomes related to negative affective and positive affective responding will also be obtained as described below. We have found that older adults show a peak onset of depressed mood at 2 hours, similar to young adults and will explore the presence of delayed termination of response in older adults; hence protocol duration will last 12 h, as compared to 6 h as was previously used in the study of adults [75]. Other secondary outcomes including assessment of physical symptoms, social dimensions, and inflammatory activity will be assessed as described in the detailed procedures below.

A between-subject parallel design will be used for the following reasons: 1) the proposed study involves endotoxin administration, and in a hypothetical crossover design, subjects who experience aversive sickness symptoms from endotoxin (possibly also from placebo due to expectation) might drop out, which would introduce bias and invalidate the results; 2) the effect of endotoxin on affective responding and inflammation might not resolve between two cross-over sessions of the experimental protocol; 3) repeated exposure to behavioral tests of affect responding might alter responses; 4) two cross-over sessions of the experimental protocol have higher subject burden.

Because the administration of endotoxin in this experimental protocol is designed to mimic the increases in plasma cytokines reported in chronic low-grade inflammatory conditions (i.e., 2-

10 fold increases in interleukin [IL-6] and tumor necrosis factor α [TNF]) [76,77], we will use low dose endotoxin (0.8 ng/kg) similar to what we have used in our prior studies [75]. An endotoxin dose of 0.8 ng/kg yields significant increases in proinflammatory cytokines, and increases in depressed mood and anhedonia. Our preliminary data indicate that older adults show similar response to endotoxin without adverse events. Other inflammatory experimental challenge models have been proposed, namely administration of interferon (IFN)-α or typhoid vaccine. However behavioral effects of IFN-α do not usually occur until 8–12 weeks of treatment [78], and typhoid vaccine induces modest increases in IL-6 that are not robustly associated with changes in depressive symptoms [79-81].

Participants

Recruitment of participants:

Given our focus on community-dwelling older adults, we employ survey-based, age-targeted sampling methods, which involve obtaining a list of telephone numbers and mailing addresses of households with at least one person aged 60 years or older from the Genesys Sampling Systems (Fort Washington, PA) or from the UCLA Clinical and Translational Science Institute (CTSI) Informatics Program. First, the Genesys Sampling System is a company that has been engaged in supporting various national surveys [82,83]. Genesys Sampling Systems maintains a bimonthly updated database of all available listed telephone households in the US within a specified area. For this study a list of 5,000 households is purchased every 3 months. Initial contact is made using UCLA Institutional Review Board (IRB) approved recruitment brochures and letters, which inform potential participants of the study (i.e., a research study to examine "Sleep and Healthy Aging Research in Depression," SHARE-D) and invite them to call a hotline number if interested. A follow-up phone call is made to confirm receipt of the letter, and a phone eligibility survey is completed if the participant is interested. This two-step method

increases overall response rate and reduces interviewer time by 80% compared to a random-digit-dial sample surveys [83]. Second, we obtained listing of persons aged 60 to 80 years who were enlisted in the UCLA Healthcare System, using the UCLA CTSI Informatics Program. This program queries xDR – a clinical data warehouse system containing data from UCLA's CareConnect (Epic) Electronic Health Records (HER) linked with to older adult legacy systems and other sources. In addition to age-targeted sampling methods, the UCLA CTSI Informatics Program identifies those persons who have expressed interest in participating in research. Data from the UCLA CTSI Informatics Program are extracted every 6 months, including demographic and contact information. As noted above, participants are first contacted by brochure and letters, with follow-up phone contact. The survey sample for Genesys Sampling Systems and the CTSI Informatics Program are limited to households living within a 15 mile radius of the UCLA Westwood Campus, given the logistics of assessment and transport to UCLA for baseline assessment and the experimental protocol. We have employed sampling methods similar to those described for other randomized controlled trials that have enrolled older adults with insomnia as previously reported [9,64,84-86].

Eligibility Criteria

Inclusion Criteria: Participants will be required to be in good general health (as evaluated during eligibility assessment by phone and in-person interview); aged 60 to 80 years old; those with insomnia disorder will be identified by the Structured Clinical Interview for DSM-5 (SCID-DSM-5)[87]American Psychiatric Association., 2013 #11473} and the Duke Structured Interview for Sleep Disorders (DSISD)[88]. Comparison controls are those who do not fulfill diagnostic criteria for insomnia. Whereas short sleep duration along with insomnia are thought to have greater biological impact than insomnia alone [89-91], the broad goal of this research is to inform understanding of the mechanisms linking insomnia to depression. Inclusion of only those with insomnia and short sleep duration would limit generalizability. Further, because the diagnosis of

insomnia does not employ objective sleep assessment such as actigraphy or polysomnography to evaluate the nature and severity of insomnia complaints, neither of these methods will be used to determine the presence or absence of DSM-5 insomnia disorder. Nevertheless, we will assess the duration and severity of insomnia, and participants will complete 14 days of sleep diary and actigraphy to evaluate sleep duration and sleep efficiency as described below.

Exclusion Criteria: The following medical conditions result in exclusion: presence of chronic physical illnesses; history of allergies, autoimmune, liver, or other severe chronic diseases; current or history (last 6 months) of medical conditions not limited to but including cardiovascular (e.g., history of acute coronary event, stroke) and neurological diseases (e.g., Parkinson's disease), as well as pain disorders; inflammatory disorders (e.g., rheumatoid arthritis) or other autoimmune disorders; uncontrolled medical conditions that are deemed by the investigators to interfere with the proposed study procedures, or to put the study participant at undue risk; chronic infection, which may elevate proinflammatory cytokines; acute infectious illnesses within the two weeks prior of the experimental session.

The following psychiatric and sleep disorders will result in exclusion: current Axis I psychiatric disorders as determined by SCID-5 including a current major depressive disorder and substance dependence; history of depression within last year, although history of depression greater than one year prior to enrollment will not be an exclusion criterion (a pre-planned sensitivity analysis will evaluate differences in depressed mood responses and negative- and positive affective responding as a function of past history of depression); lifetime history of suicide attempt or inpatient psychiatric admission; sleep apnea as screened with the Berlin Sleep Questionnaire and further assessed with overnight sleep monitoring using the WatchPat (i.e., apnea hypoxia index >15)[92]; and phase-shift disorder as identified by the SCID-5 and the DISD or history of nightshift work or time zone shifts (> 3hrs) within the previous 6 weeks. Persons with sleep apnea are excluded given limited evidence that sleep apnea is a

prospective risk factor for depression and that prevalence rates of depression are not elevated in those with sleep apnea as compared to those without sleep apnea [93].

Additional exclusion criteria are: current and regular use of prescription medications such as steroids, non-steroid anti-inflammatory drugs, aspirin, immune-modifying drugs, opioid analgesics, statins, antihypertensive or other cardiovascular drugs (i.e., antiarrhythmic, antianginal, and anticoagulant drugs); antidepressant medications or other psychotropic medication in the last 6 months; current smoking or excessive caffeine use (>600 mg/day) because of the known effects on proinflammatory cytokine levels [94]; history of recreational drug use in last 6 months or evidence of such use as determined by screening urine test for substances; body mass index > 35 kg/m^2 because of the effects of obesity on proinflammatory cytokine activity [94] and also on risk for sleep disordered breathing; any clinically significant abnormalities on laboratory tests; clinically significant abnormalities in electrocardiogram; and evidence of cognitive impairment with scores on Mini-Mental Status Examination 24 or less. On the day of the experimental protocol, participants will be excluded should they show any of the following physical signs: blood pressure less than 90/60 or greater than 160/120 mmHG, pulse less than 50 beats/minute, or temperature greater than 99.5°F.

Trial Procedures

<u>Assessments prior to experimental protocol</u>

Screening Eligibility: Individuals with cognitive impairment or limited English proficiency, as identified at the onset of telephone contact, will not undergo screening phone interview. After verbal consent for a screening interview, interviewers will conduct an approximate 20-minute survey using a scripted telephone interview format as previously described [95] to assess demographic information and screen for the presence of insomnia or not using the Pittsburgh Sleep Quality Index (PSQI)[96]. Eligibility questions will also evaluate whether current depression is absent and exclude subjects who answer affirmatively to the following two screening

question: “Are you depressed nearly every day for two weeks or more?” and/or “Have you lost interest in normal activities nearly every day for two weeks or more?”, using items from the 10-item Center for Epidemiologic Studies Depression [97]. If subjects do not evidence symptoms of current depression, they will be advanced to interview assessment, in which the SCID-5 will be administered to confirm the absence of current depression using DSM-5 diagnostic criteria [87,98]. In addition, we will screen for ongoing medical conditions and current use of medications such as antidepressant medications.

Baseline eligibility: At the onset of this interview assessment, written informed consent is obtained after reviewing any study-related questions. This baseline eligibility assessment is a face-to-face interview format lasting about 120 minutes, including the following assessments: SCID-DSM-5 (those with current psychiatric disorder or depression in last year will be excluded) and the DSISD [88]; demographic information and medical / medication histories including recent (last two weeks) infection and Charlson Co-Morbidity Index [99] and Chronic Disease Scale[100,101]; substance use history; Mini-Mental Status Examination (those who score 24 or less will be excluded) [102]; height and body weight for calculation of body mass index, vital signs, blood sampling for screening laboratory tests (i.e., complete blood cell count, comprehensive metabolic panel, hemoglobin A1c, and Free T4 Index), and electrocardiogram (those will clinically significant abnormalities will be excluded).

A wrist actigraph and instructions for completing sleep diaries for a two-week period to monitor sleep wake patterns will be reviewed. Additionally, a Watch-PAT device will be worn for one night (those with sleep apnea and nocturnal myoclonus will be excluded) [92].

Baseline assessment of clinical and other background variables. The following baseline measures are assessed prior to the experimental protocol, but not repeatedly during the experimental protocol. Measures repeatedly assessed during the experimental protocol are described in the “Outcomes” section below.

Insomnia assessment: In addition to diagnostic evaluation of the presence of current DSM-5 insomnia disorder or not, duration and lifetime history of insomnia will be evaluated; profile of inflammatory activation may be related to duration of insomnia. Current severity of insomnia complaints will be evaluated by the Insomnia Severity Index (ISI) [103]) and the PSQI [96,104]. These self-report data are in addition to the daily sleep diary [105] as completed by an Online Sleep Diary System, accessible via computer interface with unique user IDs, and objective assessment of sleep behaviors and sleep duration by wrist actigraphy. Sleep apnea will be screened by the Berlin Questionnaire [106] with objective evaluation using the WatchPat. [92]. Sleep wake schedule will be evaluated using the Munich Chronotype Questionnaire [107] and daytime dysfunction associated with insomnia is assessed by the Fatigue Symptom Inventory (FSI) [108] and Multidimensional Fatigue Symptom Inventory-Short Form (MFSI-SF) [109].

Depression assessment: The SCID-5 interview will be used to determine the presence of past history of depression, and the absence of current depressive disorder and other psychiatric diagnoses. For depression history, we will identify number of episodes, age of onset, last episode, and treatment variables. Administration of the SCID is performed by interviewers who are trained to criterion validity; diagnoses is determined in a weekly consensus meeting to maintain reliability and criterion validity. Depressive/anxiety symptom severity is assessed at only baseline using the Patient Health Questionnaire-9 [110], Beck Depression Inventory-II [111,112], Beck Anxiety Inventory [113], General Anxiety Disorder-7 [114], and Inventory for Depressive Symptoms-Self Report [115].

Social domain: Social support measures include Social Provision Scale [116], Experiences in Close Relationships Questionnaire [117], and Interpersonal Support Evaluation List [118].

Psychosocial stress: Psychosocial stress is evaluated by Perceived Stress Scale [119], and childhood adverse psychosocial stress is evaluated using the Risky Families Questionnaire [120].

Health factors: Health variables, in addition to eligibility, include level of physical activity (i.e., Godin Leisure-Time Exercise Questionnaire) [121] and health functioning (i.e., Medical Outcomes Study Short-form (SF-36) [122].

Endotoxin vs. placebo administration methods

Following baseline assessments, inclusion and exclusion criteria will be examined and confirmed by the study physician (Michael R. Irwin, MD). The study is conducted at the UCLA Clinical Translational Research Center (CTRC). Beginning at 8 am, a CTRC nurse, blind to the randomization schedule, will assess height and weight as well as vital signs (blood pressure, pulse, temperature). As noted above, participants will be excluded if: (a) blood pressure is less than 90/60 mmHG or greater than 160/120 mmHG, (b) pulse is less than 50 beats/minute, or (c) temperature is greater than 99.5°F. An indwelling venous catheter with a heparin lock will be inserted into the participant’s dominant forearm for hourly blood draws and one into the nondominant forearm for a continuous saline flush (150 cc/h) for endotoxin vs. placebo administration. Baseline assessment of outcomes including self-report questionnaires and experimental affective response tasks will then be completed, followed by baseline blood sampling which is obtained about 60 minutes after placement of catheter and 30 minutes after completion of questionnaires and tasks. The CTRC pharmacy will receive the randomization assignment and prepare the endotoxin vs. placebo. After 90 minutes following arrival at the CTRC, participants will randomly receive either low-dose endotoxin (0.8 ng/kg of body weight) or placebo as an intravenous bolus over 30-60 seconds. NIH will provide reference endotoxin humans (E. coli group O:113). Throughout the study protocol lasting up to 12 hours, vital signs and blood sampling will be obtained every half hour for the first one hour, and then hourly thereafter. Placement of an intravenous catheter for the duration of the day has not been found to induce nonspecific increases in circulating levels of IL-6 or TNF [16].

Randomization and allocation concealment

Randomization sequence will be generated via computerized random number generator in each group for endotoxin vs. placebo in a 1:1 ratio by the study biostatistician (RO) who has no contact with participants. Allocation concealment will be maintained confidential delivery of a secure and encrypted email to the CTRC pharmacy.

Blinding

Participants will be aware that they are assigned to either endotoxin or placebo as the consent form states that participants would be assigned at random to either endotoxin or placebo as experimental conditions. Participants are blind to condition assignment, and they are also blind to the primary outcome of the study, namely depressed mood and depressive symptoms. Investigators and outcome assessors are blind to allocation.

OUTCOMES

Primary outcome:

The primary outcome is self- and observer-rated assessment of depressed mood (primary outcome) as measured by the POMS [123-125] and observer-rated depressed mood and depressive symptom severity as measured by the MADRS [126] in response to endotoxin (0.8 ng/kg body weight) vs. placebo with repeated assessment over up to 12 hours. Both the POMS and MADRS have been found to be sensitive to acute changes in depressed mood following endotoxin [70,127].

Secondary outcomes:

Secondary outcome: severity of depressive symptoms. Change in severity of depressive symptoms is assessed by the observer-rater administration of the Hamilton Rating Scale for

Depression (HAMD) [128,129], modified for administration during an acute time period with repeated assessment over up to 7 hours.

Secondary outcome: negative affective responding. Two tasks evaluate changes in negative affective responding: Emotion Recognition Task and Emotion Intensity Task, which are administered at baseline and again about 3 hours after endotoxin vs. placebo.

The Emotion Recognition Task evaluates the ability of participants to recognize an expressed facial emotion, and to rate their certainty in this choice [130,131]. Delayed recognition of a sad emotion, for example, indicates a reduced sensitivity to sad facial expressions. Facial images are taken from video-recordings of the Cohn-Kanade facial expression database (Kanade et al., 2000; Lucey et al., 2010). Images represent a range of age, sex, and ethnicity, which are distributed equally across the emotions being assessed. In total, 7 trials are used, each comprising 10 images. Within each trial, the 10 images gradually progress from a neutral expression to a specific emotion. The trials (i.e., happy, sad, angry, afraid, surprised, and neutral) are presented in a random order. After each image, which is shown as 3-sec stimuli, participants are asked to identify the presented emotion (i.e., "Which emotion is this person feeling right now?"), and answers are collected during a 10-sec response (i.e., happy, sad, angry, afraid, surprised, or nothing yet). Task performance is indexed by the number of elapsed images (i.e., delay) required to correctly identify a contiguous sequence of images, with more elapsed images (i.e., longer delays) indicating attenuated or impaired facial emotion processing. A contiguous sequence was defined as a continuous series of consistently correct answers that led up to the full emotion expression (e.g., if a participant gave a correct answer for image #1 and #2, then an incorrect answer for image #3 to #6, and again a correct answer for image #7 to #10, the delay for this trial was determined as "7", because the onset of the contiguous sequence that led up to the full emotion expression started at image #7). This analytical approach has the advantage of providing more stringent recognition criteria, as opposed to capturing impulsive responding. Whereas it is known that sleep disturbance is associated with

subjective reports of negative affect, there are few empirical studies that have examined tasks of emotional processing, even though accurate face judgments may modulate emotional reactivity. We have previously found that sleep disturbance is associated with a delay in recognition of sad facial emotion in older adults [132].

The Emotion Intensity Task evaluates subjective ratings of perceived intensity in response to various degrees of facially-expressed sadness, happiness, and anger [133] taken from NimStim set of facial affects [134] and includes sad, happy, and angry and a neutral face of one white male individual. Each emotion image morphes with the neutral image using a face-morph software (Morph 2.5), resulting in 10 images covering a range of emotion expression (i.e., image #1 reflects 10% emotion expression, image #2 reflects 20% emotion expression, etc.). In total, 10 separate images are created for each emotional category, which represents the full range of emotion expression. The trials (i.e., sadness, happiness, anger) are presented in a random order. Prior to each trial, participants are informed which specific emotion they are about to rate for intensity (e.g., "You are about to see a sad face"), and are familiarized with the full range of emotion expression. During the task itself, each emotion is presented in a separate trial, and within each trial, the 10 images were presented in a random order as 2-sec stimuli. During the three trials, participants are presented with a morphing image and are asked to identify the emotion as soon as they recognize which emotion being depicted. The image gradually progresses from neutral to the specific emotion. The participant is presented with a red "x" if the incorrect emotion is chosen. Task performance is indexed by the mean rating across the 10 images within each trial, with lower ratings indicating lower perceived intensity. Hence, Emotion Intensity Task differs from the Emotion Recognition Task; the Emotion Intensity Task evaluated the perceived intensity to a known emotion, whereas Emotion Recognition Task evaluates the ability to recognize or identify an emotion.

Secondary outcome: positive affective responding. Two tasks will be used to evaluate changes in positive affective responding: Probabilistic Reward Task (PRT) and the Effort

Expenditure for Rewards Task (EEfRT), which are administered about 2.5 hours after endotoxin vs. placebo.

The PRT is a laboratory based probabilistic reward task that objectively measures participants' ability to modulate behavior as a function of reward [135]. This task has been found to identify reduced reward learning in depressed patients which is state-dependent and also to predict the persistent diagnosis of depression in the midst of treatment [135-137]. The method [138] of this computerized reward-learning task is extensively described [135,139]. This task was selected because anhedonia is a core feature of major depressive disorder and includes a reduction in experienced pleasure (liking reward) and dysfunction in reward anticipation and reward learning [135-137]. We have previously found that an inflammatory challenge reduces neural sensitivity to reward anticipation [18]. There are limited data on the relationship between insomnia on reward responsiveness, although individuals with insomnia show reduced positive affect using ecological momentary assessment [140].

The Effort Expenditure for Rewards Task (EEfRT) is used to evaluate reward processing [141]. The EEfRT is a computerized task that assesses effort-based decision making in the context of monetary reward. During the task, participants are presented with a series of trials in which they choose between an easy, low effort trial (worth a low reward amount of $1.00) and a hard, high effort trial (worth higher reward amounts ranging between $1.24-$4.30). Easy trials required 30 button presses using the index finger of the non-dominant hand in 7 seconds, while hard trials required 100 button presses with the pinky finger of the dominant hand in 21 seconds. Participants are told that not all successfully completed trials are rewarded, and the probability that a successful response yields a reward (12%, 50%, 88%) is presented for each trial. In the current study, the EEfRT is shortened from 20 minutes to 10 minutes and hard trials used the pinky finger of the dominant hand rather than the non-dominant hand to accommodate constraints in the laboratory environment [142]. Motivation for reward on the EEfRT is operationalized by willingness to exert effort for monetary reward; i.e., the selection of high

effort/high reward trials relative to the selection of low effort/low reward trials. Sensitivity to reward is operationalized by the association between changes in monetary reward magnitude (ranging from $1.24-$4.30) and changes in likelihood of selecting high effort/high reward trials vs. low effort/low reward trials.

Other secondary outcomes related to positive affective responding include measures of interest in activities and reward responsiveness, as well as responding to social reward. Three self-report questionnaires are used to evaluate interest in activities, reward responsiveness, or hedonic experience, including Snaith-Hamilton Pleasure Scale (SHAPS) [143], and the Temporal Experience of Pleasure Scale,[144]. These questionnaires are administered at baseline and about 4 to 5 hours after endotoxin vs. placebo.

To evaluate social reward, we use a social reward task (i.e., Emotional Dot Probe task with happy faces) [138] and a questionnaire (i.e., Close Other Social Reward) [145,146] to evaluate change in social reward before and after inflammatory challenge with endotoxin vs. placebo. We have previously found that inflammatory challenge reduces reward activation to non-social reward cues, but increases activation to social reward [145,146]. The task is administered at baseline and about 1.5 hour after endotoxin vs. placebo, and the questionnaire is administered at baseline and 4 hours after endotoxin vs. placebo.

Secondary outcome: social domain Because social factors contribute to depression in part by changes inflammatory mechanisms [12], this study examines several aspects related to social connection and loneliness, social support, and sensitivity to social rejection. Perceived social connection is evaluated at baseline and repeatedly for up to 12 hours, whereas other measures are obtained at baseline and up to 5 hours after endotoxin vs. placebo.

Perceived social connection is evaluated with several self-report questionnaires including How I Feel Right Now, which includes Feelings of Social Disconnection [17], and the 10-item Revised UCLA Loneliness Scale [147].

Perceived social support is characterized by the Social Support Questionnaire Scale [148], Two-way Social Support Questionnaire [149], the Attachment Style Questionnaire [150], Social Provision Scale [116], and Experiences in Close Relationships Questionnaire [117].There is evidence that perceived social support, social connection, and social reward change in response to endotoxin [146,151].

Perceived social status is evaluated the using MacArthur Scale of Subjective Social Status (i.e., Social Ladder) [152].

Sensitivity to social rejection is examined by Fear of Negative Evaluation Scale [153,154] and the Rejection Sensitivity Scale [155]. During the experimental protocol, we also administer a behavioral task, the Cyberball Social Exclusion Task [16]. This task is administered only once at 2 hours after endotoxin vs. placebo, because it involves deception; no other real participants are engaged in this task as participants are led to believe prior to the task.

Secondary outcome: cognitive processing. Inflammatory challenge acutely alters measures of cognitive performance, including spatial learning and memory [156]. We will use the virtual Morris Water Maze task [156] to assess computerized spatial learning and memory, followed by a Room Reconstruction Task [157] to assess the ability of cognitive mapping under real-life conditions. In addition, we assess several measures of cognitive processing and executive functioning, including the Cognitive Testing Stress Scale, the Spatial 2-Back Test, the Color Shape Task [158] and the Anti-saccade Task [159]. The virtual Morris Water Maze and the Room Reconstruction Task are administered 2 hours following administration of endotoxin vs. placebo. Other cognitive measures are administered at baseline and 4 hours following administration of endotoxin vs. placebo.

Secondary outcome: physical symptoms. The Brief Symptom Inventory [160], which assesses a variety of somatic symptoms related to psychological distress is administered up to 5 hours following administration of endotoxin vs. placebo. Physical “sickness” symptoms which have been related to the administration of endotoxin are assessed using the Physical Symptom

Questionnaire as described [15,17]. These physical or “sickness symptoms” include self-reported rating of severity of muscle pain, shivering, nausea, breathing, difficulties, and fatigue are self-rated up to 12 hours.

Secondary outcome: markers of inflammatory response. Circulating levels of pro- and anti-inflammatory cytokines including IL-6, TNF, IL-8, IL-10 and IFNγ are evaluated at baseline prior to the infusion, 30 and 60 minutes post infusion, and hourly for the remainder of the experimental protocol. Baseline levels of inflammation as indexed by CRP, and Toll-like receptor (TLR)-4 stimulated monocyte production of IL-6 and TNF, may be related to inflammatory responsivity to challenge with endotoxin vs. placebo (i.e., inflammatory priming of the endotoxin response), and these measures will be obtained at baseline. We also examine upstream pathways related to activation of inflammatory cytokines using whole blood samples collected at baseline and 30, 60, and 120 minutes after infusion; RNA samples will be used to evaluate expression of genes involved in proinflammatory pathways (IL1B, IL6, IL8, CD83, CCL3, TNFAIP3, and NF-κB/Rel family) using quantitative real-time RT-PCR using established TaqMan Gene Expression Assays with transcriptional profiling of the Conserved Translational Response to Adversity (CTRA) in circulating peripheral blood mononuclear cells (PBMCs) [161,162].

Safety monitoring plan

We have extensive experience with all aspects of the endotoxin vs. placebo inflammatory challenge and have administered endotoxin dose 0.8 ng/kg in over 150 adult participants (20-65 y) with no evidence of adverse events (AEs). Other studies have found that endotoxin dose 0.8 ng/kg can be administered in older adult subjects with no adverse effects [77]. However, a 2.5-fold increase in the endotoxin dose to 2.0 ng/kg results in greater increases in proinflammatory cytokines, fever responses, and hypotension in older adults (61-69 years) as compared to young adults (20-27 years, unpublished data).

Safety monitoring is actively conducted throughout the study with hourly questions about physical symptoms and monitoring of vital signs. In addition, participants undergo a phone interview 1 and 7 days after the experimental session, in which there is active querying about physical symptoms, and evaluation of depressive symptoms by the MADRS. Therefore, adverse events will be identified by continuous reporting, and reviewed by the Principal Investigator (MRI).

Participants are given a 24-hour telephone number for calling the physician should feelings of depression or suicidal thoughts emerge in the 7 days following the protocol. In the event that significant medical or psychiatric problems are encountered, the study blind will be broken so that appropriate medical treatment will be provided.

The Principal Investigator has also designated appropriately qualified personnel to periodically perform quality assurance checks during and after the study. Such monitoring provides the opportunity to evaluate the progress of the study and to obtain information about potential problems. The monitor will assure that data are accurate and in agreement with any paper source documentation used, verify that subjects' consent for study participation has been properly obtained and documented, confirm that research subjects entered into the study meet inclusion and exclusion criteria, verify that study procedures are being conducted according to the protocol guidelines, monitor review AEs and serious adverse events (SAEs), and assure that all essential documentation required by Good Clinical Practices (GCP) guidelines are appropriately filed. At the end of the study, the monitors will confirm that the site has the appropriate essential documents on file, and advise on storage of study records.

An independent Data and Safety Monitoring Board (DSMB) is scheduled to meet every 6 months for the duration of the study. The DSMB is blind to subjects' actual randomized group assignments but has the opportunity to request at any time that the blind be broken, if concerns arise from the blinded data. Ad hoc meetings will be convened if SAEs occur that are

considered at least possibly related to the study procedures. For any adverse event, the DMSB determines whether the event is related to experimental protocol if it occurred during the experimental protocol, or if it could be attributed to the protocol if it occurred during the 7-day follow-up.

Ethics

Institutional Review Board Review,

The study will be conducted under a protocol as reviewed by the UCLA IRB; the study is conducted by scientifically and medically qualified persons; the benefits of the study are in proportion to the risks; the rights and welfare of the subjects are respected; the physicians conducting the study ensure that the hazards do not outweigh the potential benefits; the results reported will be accurate; subjects give their informed consent and are competent to do so and not under duress; and all study staff comply with the ethical principles in 21 Code of Federal Regulations (CFR) Part 50 and the Belmont Principles.

Ethical Conduct of the Study

This study is conducted in accordance with all applicable Federal human research protections requirements and the Belmont Principles of respect for persons, beneficence, and justice. The procedures set out in this study are designed to ensure that all study personnel abide by the principles of the ICH GCP Guideline and the Code of Federal Regulations (CFR). The PI confirms this by signing FDA Form 1572.

Confidentiality of Data and Subject Records

To maintain subject confidentiality, all laboratory specimens, eCRFs, reports and other records are identified by a subject number only. Research and clinical records are stored in a locked cabinet. Only research staff, and other required regulatory representatives have access to the records. Subject information is not released without written permission.

Compensation for Participation

Subjects are compensated for travel expenses and for time contributed to this research study in the form of cash. Compensation is provided at each subject visit and is detailed in the informed consent form.

Written Informed Consent

The informed consent process and documents were reviewed and approved by the IRB and prior to initiation of the study. The IRB approved the screening eligibility scripts and the verbal consenting process for the screening interview. The IRB also approved the written consent document that contained a full explanation of the possible risks, advantages, and alternate treatment options, and availability of treatment in the case of injury, in accordance with 21 CFR Part 50. The consent document indicates that by signature, the subject. A written informed consent document, in compliance with 21 CFR Part 50, 32 CFR Part 219, and the Belmont Principles, and HIPAA Authorization is signed by the subject before any study-related procedures are initiated for each subject. All potential subjects for the study are given a current copy of the Informed Consent Form to read. All aspects of the study and informed consent are explained in lay language to the subject by either the investigator, or a medically trained designee. Any subject who is unable to demonstrate understanding of the information contained in the informed consent will be excluded from study participation.

All study subjects are given a copy of the signed informed consent.

Data Handling and Record Keeping

Source documents include but are not limited to original documents, data and records such as interview data, questionnaire data, and laboratory results. Data are transcribed from source documentation directly into a statistical program (i.e., SPSS). Paper copies of interview and questionnaire are available. The transcribed data are consistent with the source documents or the discrepancies are explained with a note in the source document. All entries, corrections, and alterations are made by the investigator or other authorized study personnel.

Subject Identification and Confidentiality

Subjects are identified by unique study ID numbers, and all paper source documents use this a unique subject number. No personal identifier will be used in any publication or communication used to support this research study. The subject number is used if it becomes necessary to identify data specific to a single subject. Regulatory bodies, such as the IRB, are eligible to review research records related to this study as a part of their responsibility to protect human subjects in clinical research. Personal identifiers are removed from research records.

Retention of Records

The investigator is responsible for creating and/or maintaining all study documentation required by Title 21 Code of Federal Regulations (21CFR) Parts 50, 54, 56, and 312, ICH E6 section 8, as well as any other documentation defined in the protocol. Federal and local regulations require that the investigator retain a copy of all regulatory documents and records that support the data for this study for at least 5 years following the date on which the results of the investigation were submitted for scientific publication and/or reported on clinicaltrials.gov.

Data Sharing Plan

The study include data from older adults with and without insomnia. The final dataset will include self-reported demographic and behavioral data from interviews, behavioral tasks, laboratory data from blood samples that characterize cellular and genomic markers of inflammation. The final dataset is stripped of identifiers prior to release for sharing, but there remains the possibility of deductive disclosure of subjects with unusual characteristics. Thus, we will make the data and associated documentation available to users only under a data-sharing agreement that provides for: (1) a commitment to using the data only for research purposes and not to identify any individual participant; (2) a commitment to securing the data using appropriate computer technology; (3) a commitment to destroying or returning the data after analyses are completed; 4) complies with UCLA IRB protocols for protected health information of its members; and 5) commitment to testing of a priori hypotheses. The timing of sharing of data occurs no later than acceptance for publication of the main findings of this project.

Statistical Methods

Sample size

Data from several studies show that endotoxin induces increases in depressed mood as measured by the POMS with a moderate to large effects (d=.48 to 85) [15-17]. In addition, endotoxin induces an exaggerated increase in depressed mood in adults with modest sleep disturbance, as compared to those without sleep disturbance, with a large effect (d=.71)[19]. Using these preliminary data, Monte Carlo simulations indicate that sample sizes ranging from 42 to 60 yield a minimum of 85% power (α=.05, two-tailed) for within time comparisons of main effect of condition (i.e., endotoxin vs. placebo), main effect of group (i.e., insomnia vs. comparison controls), and the interaction of condition x group, assuming that the interaction effect in older adults is similar to effects previously reported in adults [19]. Hence, a target sample size of n= 60 per condition (i.e., endotoxin, placebo) or n=60 per group (i.e., insomnia, comparison control) achieves 85% power for main effect of condition, main effect of group, and their interaction. Omnibus linear mixed models LMM analysis of repeated measures of depressed mood (primary outcome) yield greater statistical power (>90%). In addition, oversampling of the comparison controls, per protocol, yields even greater statistical power for between comparisons of group (i.e., insomnia, n=60; comparison control, n=100) and condition (i.e., endotoxin, n=80; placebo, n=80), and within group (i.e., control).

Statistical analysis

Analyses will be reported according to the CONSORT statement, and will use Intent-To-Treat samples (i.e., all persons who were randomized and received either endotoxin or placebo will be included in the analysis). Measured baseline and outcome variables will be assessed for distributional qualities and transformed if necessary for use in the selected statistical models. All pre-classification variables, including age (60-70 years vs. 71-80 years), sex, and body mass

index (18-24.9 vs. 25–35 kg/m^2), as well as other baseline values will be compared between healthy controls and insomnia patients; those having a relationship with the outcome variables will be considered for inclusion as covariates in the main analyses. The detailed statistical analysis plan will be reviewed by the independent Trial Data Monitoring and Steering Committees of the UCLA Clinical Translational Sciences Institute. IBM SPSS Version 28, SAS version 9.4 or other established statistical packages will be used for all analyses.

The basic design is a 2 group (insomnia vs. comparison controls) by 2 condition (endotoxin vs. placebo), analysis variance (ANOVA) with one or more repeated measures of outcome analyzed with linear mixed models (LMM). LMM provides unbiased estimates when observations are missing at random. The timing repeated measures varies by assessment as noted above. The key results are the main effects of group, condition, and their interaction; for those analyses with repeated measures, the two main effects and interaction will further interact with the time variable.

Primary Outcome: Depressed Mood. The primary outcome is tested with LMM for the depressed mood subscale of the POMS and for severity of depressed mood and depressive symptoms of the MADRS with a time frame up to 12 hours.

Secondary Outcome: Depressive Symptoms. Change in depressive symptoms is tested with LMM for the clinician-rated assessment of depressive symptom severity as measured Hamilton Rating Scale for Depression with a time frame up to 8 hours.

Secondary Outcome: Negative Affective Responding. Change in negative affective responding is tested with LMM for the following behavioral tasks, Emotion Recognition Task and Emotion Intensity Task with a time frame of about 2 hours. Similar analytic strategy will be

applied for other secondary outcomes related to negative affect responding with time frames ranging from 4- to 12 hours.

Secondary Outcome: Positive Affective Responding. Change in positive affective responding is is tested with LMM for the following behavioral tasks, reward learning (i.e., PRT) and reward motivation and sensitivity to monetary reward (EEfRT) with a time frame of about 2 hours

Other secondary outcomes

All other outcomes as listed above including the inflammatory cytokines are tested using LMM as described with a time frame up to 12 hours.

For the analyses of the inflammatory transcriptional profiles, we will focus upon specialized genomic analyses and utilize methods previously reported by us. Quantile-normalized gene expression values will be log2- transformed and subject to GLM analysis to provide maximum likelihood point estimates of differential transcript abundance between group (insomnia, comparsion control) and condition (endotoxin, placebo), which provide maximally replicable inputs into the higher-order set-based bioinformatics analyses. TELiS promoter-based bioinformatics analyses will test the hypothesis that PBMCs will show alterations by group and condition in global gene expression profiles consistent with decreased activity of the pro-inflammatory transcription factors NF-κB and AP-1. To identify the primary cellular sources of differentially expressed genes, we will carry out Transcript Origin Analysis. In both TELiS and Transcript Origin Analyses, standard errors will be estimated by 2000 cycles of bootstrap resampling of residual vectors from the linear models used to estimate differential gene expression across group and condition (controlling for correlated expression across genes).

Pre-specified secondary analyses

We will examine whether main condition effects differ as a function of sex, and interactions with sex for the primary and secondary outcomes will be tested within the total sample and within the groups. A pre-planned sensitivity analysis will evaluate differences in depressed mood responses and negative- and positive affective responding as a function of past history of depression. We will also examine the relation between changes in inflammatory outcomes and primary- and secondary outcomes. We and others have previously found that endotoxin vs. placebo induces robust activation of inflammatory outcomes including circulating levels of cytokines and transcriptional inflammatory gene profile [16-19,163,164]. To test whether circulating levels of cytokines are related to primary and secondary outcomes, circulating cytokines will be used as concurrent and lagged predictors of the outcomes. Additional planned exploratory analyses will test mediation. Using the methods of Hayes et al. and Hayes PROCESS macro (version 4.1) [165-168], the hypothesized mediation model is tested,[169] in which the effect of endotoxin on the primary outcome (i.e., POMS Depression) is mediated by increase in inflammation as temporally ordered mediational models will be adjusted for the above set of covariates included in the LMM analyses.

REFERENCES

1. Carney RM, Freedland KE, Miller GE, Jaffe AS. Depression as a risk factor for cardiac mortality and morbidity: a review of potential mechanisms. *J Psychosom Res.* 2002;53(4):897-902.
2. Cuijpers P, Vogelzangs N, Twisk J, Kleiboer A, Li J, Penninx BW. Differential mortality rates in major and subthreshold depression: meta-analysis of studies that measured both. *Br J Psychiatry.* 2013;202(1):22-27. doi:10.1192/bjp.bp.112.112169.
3. Alexopoulos GS. Depression in the elderly. *Lancet.* 2005;365(9475):1961-1970. doi:10.1016/S0140-6736(05)66665-2.
4. Charney DS, Nemeroff CB, Lewis L, et al. National Depressive and Manic-Depressive Association consensus statement on the use of placebo in clinical trials of mood disorders. *Arch Gen Psychiatry.* 2002;59(3):262-270. doi:yps20346 [pii].
5. Thase ME. Achieving remission and managing relapse in depression. *The Journal of clinical psychiatry.* 2003;64 Suppl 18:3-7.
6. Baglioni C, Battagliese G, Feige B, et al. Insomnia as a predictor of depression: a meta-analytic evaluation of longitudinal epidemiological studies. *J Aff Dis.* 2011;135(1-3):10-19. doi:10.1016/j.jad.2011.01.011.
7. Cho HJ, Lavretsky H, Olmstead R, Levin MJ, Oxman MN, Irwin MR. Sleep disturbance and depression recurrence in community-dwelling older adults: a prospective study. *Am J Psychiatry.* 2008;165(12):1543-1550. doi:10.1176/appi.ajp.2008.07121882.
8. Lee E, Cho HJ, Olmstead R, Levin MJ, Oxman MN, Irwin MR. Persistent sleep disturbance: a risk factor for recurrent depression in community-dwelling older adults. *Sleep.* 2013;36(11):1685-1691. doi:10.5665/sleep.3128.

9. Irwin MR, Carrillo C, Sadeghi N, Bjurstrom MF, Breen EC, Olmstead R. Prevention of incident and recurrent major depression in older adults with insomnia: a randomized clinical trial. *JAMA psychiatry.* 2022;79(1):33-41. doi:10.1001/jamapsychiatry.2021.3422.

10. Irwin MR. Sleep and inflammation: partners in sickness and in health. *Nat Rev Immunol.* 2019;19(11):702-715. doi:10.1038/s41577-019-0190-z.

11. Miller AH, Raison CL. The role of inflammation in depression: from evolutionary imperative to modern treatment target. *Nat Rev Immunol.* 2016;16(1):22-34. doi:10.1038/nri.2015.5.

12. Slavich GM, Irwin MR. From stress to inflammation and major depressive disorder: a social signal transduction theory of depression. *Psychol Bull.* 2014;140(3):774-815. doi:10.1037/a0035302.

13. Lasselin J, Lekander M, Benson S, Schedlowski M, Engler H. Sick for science: experimental endotoxemia as a translational tool to develop and test new therapies for inflammation-associated depression. *Mol Psychiatry.* 2021;26(8):3672-3683. doi:10.1038/s41380-020-00869-2.

14. Schedlowski M, Engler H, Grigoleit JS. Endotoxin-induced experimental systemic inflammation in humans: a model to disentangle immune-to-brain communication. *Brain Behav Immun.* 2014;35:1-8. doi:10.1016/j.bbi.2013.09.015.

15. Moieni M, Irwin MR, Jevtic I, Olmstead R, Breen EC, Eisenberger NI. Sex differences in depressive and socioemotional responses to an inflammatory challenge: implications for sex differences in depression. *Neuropsychopharmacology.* 2015;40(7):1709-1716. doi:10.1038/npp.2015.17.

16. Eisenberger NI, Inagaki TK, Rameson LT, Mashal NM, Irwin MR. An fMRI study of cytokine-induced depressed mood and social pain: the role of sex differences. *Neuroimage.* 2009;47(3):881-890. doi:10.1016/j.neuroimage.2009.04.040.

17. Eisenberger NI, Inagaki TK, Mashal NM, Irwin MR. Inflammation and social experience: an inflammatory challenge induces feelings of social disconnection in addition to depressed mood. *Brain Behav Immun.* 2010;24(4):558-563. doi:10.1016/j.bbi.2009.12.009.
18. Eisenberger NI, Berkman ET, Inagaki TK, Rameson LT, Mashal NM, Irwin MR. Inflammation-induced anhedonia: endotoxin reduces ventral striatum responses to reward. *Biol Psychiatry.* 2010;68(8):748-754. doi:10.1016/j.biopsych.2010.06.010.
19. Cho HJ, Eisenberger NI, Olmstead R, Breen EC, Irwin MR. Preexisting mild sleep disturbance as a vulnerability factor for inflammation-induced depressed mood: a human experimental study. *Translational psychiatry.* 2016;6(3):e750. doi:10.1038/tp.2016.23.
20. Irwin MR, Piber D. Insomnia and inflammation: a two hit model of depression risk and prevention. *World Psychiatry.* 2018;17(3):359-361. doi:10.1002/wps.20556.
21. Piber D, Olmstead R, Cho JH, et al. Inflammaging: Age and Systemic, Cellular, and Nuclear Inflammatory Biology in Older Adults. *J Gerontol A Biol Sci Med Sci.* 2019;74(11):1716-1724. doi:10.1093/gerona/glz130.
22. Kennedy BK, Berger SL, Brunet A, et al. Geroscience: linking aging to chronic disease. *Cell.* 2014;159(4):709-713. doi:10.1016/j.cell.2014.10.039.
23. Franceschi C, Campisi J. Chronic inflammation (inflammaging) and its potential contribution to age-associated diseases. *J Gerontol A Biol Sci Med Sci.* 2014;69 Suppl 1:S4-9. doi:10.1093/gerona/glu057.
24. Besedovsky L, Lange T, Haack M. The Sleep-Immune Crosstalk in Health and Disease. *Physiol Rev.* 2019;99(3):1325-1380. doi:10.1152/physrev.00010.2018.
25. Gimeno D, Kivimaki M, Brunner EJ, et al. Associations of C-reactive protein and interleukin-6 with cognitive symptoms of depression: 12-year follow-up of the Whitehall II study. *Psychol Med.* 2009;39(3):413-423. doi:S0033291708003723 [pii]

10.1017/S0033291708003723.

26. Raison CL, Miller AH. Role of inflammation in depression: implications for phenomenology, pathophysiology and treatment. *Modern trends in pharmacopsychiatry.* 2013;28:33-48. doi:10.1159/000343966.
27. Mac Giollabhui N, Ng TH, Ellman LM, Alloy LB. The longitudinal associations of inflammatory biomarkers and depression revisited: systematic review, meta-analysis, and meta-regression. *Mol Psychiatry.* 2021;26(7):3302-3314. doi:10.1038/s41380-020-00867-4.
28. Valkanova V, Ebmeier KP, Allan CL. CRP, IL-6 and depression: a systematic review and meta-analysis of longitudinal studies. *J Affect Disord.* 2013;150(3):736-744. doi:10.1016/j.jad.2013.06.004.
29. Chung HY, Cesari M, Anton S, et al. Molecular inflammation: underpinnings of aging and age-related diseases. *Ageing research reviews.* 2009;8(1):18-30. doi:10.1016/j.arr.2008.07.002.
30. Irwin MR, Olmstead R, Carrillo C, et al. Sleep loss exacerbates fatigue, depression, and pain in rheumatoid arthritis. *Sleep.* 2012;35(4):537-543. doi:10.5665/sleep.1742.
31. Pace TW, Negi LT, Sivilli TI, et al. Innate immune, neuroendocrine and behavioral responses to psychosocial stress do not predict subsequent compassion meditation practice time. *Psychoneuroendocrinology.* 2010;35(2):310-315. doi:10.1016/j.psyneuen.2009.06.008.
32. Pace TW, Mletzko TC, Alagbe O, et al. Increased stress-induced inflammatory responses in male patients with major depression and increased early life stress. *Am J Psychiatry.* 2006;163(9):1630-1633.
33. Weinstein AA, Deuster PA, Francis JL, Bonsall RW, Tracy RP, Kop WJ. Neurohormonal and inflammatory hyper-responsiveness to acute mental stress in depression. *Biol Psychol.* 2010;84(2):228-234. doi:10.1016/j.biopsycho.2010.01.016.

34. Dassonville C, Bonfils P, Momas I, Seta N. Nasal inflammation induced by a common cold: comparison between controls and patients with nasal polyposis under topical steroid therapy. *Acta otorhinolaryngologica Italica : organo ufficiale della Societa italiana di otorinolaringologia e chirurgia cervico-facciale.* 2007;27(2):78-82.

35. McIntire CR, Yeretssian G, Saleh M. Inflammasomes in infection and inflammation. *Apoptosis : an international journal on programmed cell death.* 2009;14(4):522-535. doi:10.1007/s10495-009-0312-3.

36. Aschbacher K, Adam EK, Crofford LJ, Kemeny ME, Demitrack MA, Ben-Zvi A. Linking disease symptoms and subtypes with personalized systems-based phenotypes: a proof of concept study. *Brain Behav Immun.* 2012;26(7):1047-1056. doi:10.1016/j.bbi.2012.06.002.

37. De Kock M, Loix S, Lavand'homme P. Ketamine and peripheral inflammation. *CNS Neurosci Ther.* 2013;19(6):403-410. doi:10.1111/cns.12104.

38. Wang N, Yu H-Y, Shen X-F, et al. The rapid antidepressant effect of ketamine in rats is associated with down-regulation of pro-inflammatory cytokines in the hippocampus. *Upsala journal of medical sciences.* 2015;120(4):241-248. doi:10.3109/03009734.2015.1060281.

39. Yang J, Fan C, Pan L, et al. C-reactive protein plays a marginal role in cognitive decline: a systematic review and meta-analysis. *Int J Geriatr Psychiatry.* 2015;30(2):156-165. doi:10.1002/gps.4236.

40. Roose SP, Schatzberg AF. The efficacy of antidepressants in the treatment of late-life depression. *J Clin Psychopharmacol.* 2005;25(4 Suppl 1):S1-7. doi:10.1097/01.jcp.0000162807.84570.6b.

41. Chisholm D, Sanderson K, Ayuso-Mateos JL, Saxena S. Reducing the global burden of depression: population-level analysis of intervention cost-effectiveness in 14 world regions. *Br J Psychiatry.* 2004;184:393-403.

42. Andrews G, Issakidis C, Sanderson K, Corry J, Lapsley H. Utilising survey data to inform public policy: comparison of the cost-effectiveness of treatment of ten mental disorders. *Br J Psychiatry.* 2004;184:526-533. doi:10.1192/bjp.184.6.526.

43. Cuijpers P, Reynolds CF, 3rd. Increasing the Impact of Prevention of Depression-New Opportunities. *JAMA psychiatry.* 2022;79(1):11-12. doi:10.1001/jamapsychiatry.2021.3153.

44. Mrazek P, Haggerty R. *Reducing Risks for Mental Disorders: Frontiers for Preventive Intervention Research.* Washington, D.C.: National Academy Press; 1994.

45. Medicine Io. Meeting the Psychosocial Needs of Women with Breast Cancer. *Institute of Medicine of the National Academies.* 2004.

46. Munoz RF, Cuijpers P, Smit F, Barrera AZ, Leykin Y. Prevention of major depression. *Annu Rev Clin Psychol.* 2010;6:181-212. doi:10.1146/annurev-clinpsy-033109-132040.

47. Reynolds CF, 3rd. Prevention of depressive disorders: a brave new world. *Depress Anxiety.* 2009;26(12):1062-1065. doi:10.1002/da.20644.

48. Reynolds CF, 3rd, Alexopoulos GS, Katz IR, Lebowitz BD. Chronic depression in the elderly: approaches for prevention. *Drugs Aging.* 2001;18(7):507-514.

49. Cole MG, Dendukuri N. Risk factors for depression among elderly community subjects: a systematic review and meta-analysis. *Am J Psychiatry.* 2003;160(6):1147-1156.

50. Yoo SS, Gujar N, Hu P, Jolesz FA, Walker MP. The human emotional brain without sleep--a prefrontal amygdala disconnect. *Curr Biol.* 2007;17(20):R877-878. doi:10.1016/j.cub.2007.08.007.

51. Walker MP. Sleep, memory and emotion. *Prog Brain Res.* 2010;185:49-68. doi:10.1016/B978-0-444-53702-7.00004-X.

52. Zohar D, Tzischinsky O, Epstein R, Lavie P. The effects of sleep loss on medical residents' emotional reactions to work events: a cognitive-energy model. *Sleep.* 2005;28(1):47-54.

53. Nofzinger EA, Buysse DJ, Germain A, Price JC, Miewald JM, Kupfer DJ. Functional neuroimaging evidence for hyperarousal in insomnia. *Am J Psychiatry.* 2004;161(11):2126-2128. doi:10.1176/appi.ajp.161.11.2126.
54. Bonnet MH, Arand DL. Hyperarousal and insomnia: state of the science. *Sleep Med Rev.* 2010;14(1):9-15. doi:10.1016/j.smrv.2009.05.002.
55. Riemann D, Spiegelhalder K, Feige B, et al. The hyperarousal model of insomnia: a review of the concept and its evidence. *Sleep Med Rev.* 2010;14(1):19-31. doi:10.1016/j.smrv.2009.04.002.
56. Morin CM, Bootzin RR, Buysse DJ, Edinger JD, Espie CA, Lichstein KL. Psychological and behavioral treatment of insomnia:update of the recent evidence (1998-2004). *Sleep.* 2006;29(11):1398-1414.
57. Vgontzas AN, Papanicolaou DA, Bixler EO, et al. Circadian interleukin-6 secretion and quantity and depth of sleep. *J Clin Endocrinol Metab.* 1999;84(8):2603-2607.
58. Shearer WT, Reuben JM, Mullington JM, et al. Soluble TNF-alpha receptor 1 and IL-6 plasma levels in humans subjected to the sleep deprivation model of spaceflight. *J Allergy Clin Immunol.* 2001;107(1):165-170. doi:10.1067/mai.2001.112270.
59. Redwine L, Dang J, Hall M, Irwin M. Disordered sleep, nocturnal cytokines, and immunity in alcoholics. *Psychosom Med.* 2003;65(1):75-85.
60. Irwin MR. Why sleep is important for health: a psychoneuroimmunology perspective. *Annu Rev Psychol.* 2015;66:143-172. doi:10.1146/annurev-psych-010213-115205.
61. Irwin MR, Wang M, Ribeiro D, et al. Sleep loss activates cellular inflammatory signaling. *Biol Psychiatry.* 2008;64(6):538-540. doi:10.1016/j.biopsych.2008.05.004.
62. Irwin MR, Wang M, Campomayor CO, Collado-Hidalgo A, Cole S. Sleep deprivation and activation of morning levels of cellular and genomic markers of inflammation. *Arch Intern Med.* 2006;166(16):1756-1762. doi:10.1001/archinte.166.16.1756.

63. Irwin MR, Witarama T, Caudill M, Olmstead R, Breen EC. Sleep loss activates cellular inflammation and signal transducer and activator of transcription (STAT) family proteins in humans. *Brain Behav Immun.* 2015;47:86-92. doi:10.1016/j.bbi.2014.09.017.

64. Irwin MR, Olmstead R, Carrillo C, et al. Cognitive behavioral therapy vs. Tai Chi for late life insomnia and inflammatory risk: a randomized controlled comparative efficacy trial. *Sleep.* 2014;37(9):1543-1552. doi:10.5665/sleep.4008.

65. Janicki-Deverts D, Cohen S, Doyle WJ, Turner RB, Treanor JJ. Infection-induced proinflammatory cytokines are associated with decreases in positive affect, but not increases in negative affect. *Brain Behav Immun.* 2007;21(3):301-307. doi:10.1016/j.bbi.2006.09.002.

66. Bucks RS, Gidron Y, Harris P, Teeling J, Wesnes KA, Perry VH. Selective effects of upper respiratory tract infection on cognition, mood and emotion processing: a prospective study. *Brain Behav Immun.* 2008;22(3):399-407. doi:10.1016/j.bbi.2007.09.005.

67. Motivala SJ, Khanna D, FitzGerald J, Irwin MR. Stress activation of cellular markers of inflammation in rheumatoid arthritis: protective effects of tumor necrosis factor alpha antagonists. *Arthritis Rheum.* 2008;58(2):376-383.

68. Wang N, Yu HY, Shen XF, et al. The rapid antidepressant effect of ketamine in rats is associated with down-regulation of pro-inflammatory cytokines in the hippocampus. *Upsala journal of medical sciences.* 2015;120(4):241-248. doi:10.3109/03009734.2015.1060281.

69. Yang JJ, Wang N, Yang C, Shi JY, Yu HY, Hashimoto K. Serum interleukin-6 is a predictive biomarker for ketamine's antidepressant effect in treatment-resistant patients with major depression. *Biol Psychiatry.* 2015;77(3):e19-20. doi:10.1016/j.biopsych.2014.06.021.

70. DellaGioia N, Hannestad J. A critical review of human endotoxin administration as an experimental paradigm of depression. *Neurosci Biobehav Rev.* 2010;34(1):130-143. doi:10.1016/j.neubiorev.2009.07.014.

71. Insel TR. The NIMH Research Domain Criteria (RDoC) Project: precision medicine for psychiatry. *Am J Psychiatry.* 2014;171(4):395-397. doi:10.1176/appi.ajp.2014.14020138.
72. Insel T, Cuthbert B, Garvey M, et al. Research domain criteria (RDoC): toward a new classification framework for research on mental disorders. *Am J Psychiatry.* 2010;167(7):748-751. doi:10.1176/appi.ajp.2010.09091379.
73. Hannestad J, DellaGioia N, Ortiz N, Pittman B, Bhagwagar Z. Citalopram reduces endotoxin-induced fatigue. *Brain Behav Immun.* 2011;25(2):256-259. doi:10.1016/j.bbi.2010.10.013.
74. Scott NW, McPherson GC, Ramsay CR, Campbell MK. The method of minimization for allocation to clinical trials. a review. *Control Clin Trials.* 2002;23(6):662-674. doi:10.1016/s0197-2456(02)00242-8.
75. Moieni M, Tan KM, Inagaki TK, et al. Sex Differences in the Relationship Between Inflammation and Reward Sensitivity: A Randomized Controlled Trial of Endotoxin. *Biol Psychiatry Cogn Neurosci Neuroimaging.* 2019;4(7):619-626. doi:10.1016/j.bpsc.2019.03.010.
76. Suffredini AF, Noveck RJ. Human endotoxin administration as an experimental model in drug development. *Clin Pharmacol Ther.* 2014;96(4):418-422. doi:10.1038/clpt.2014.146.
77. Suffredini AF, Hochstein HD, McMahon FG. Dose-related inflammatory effects of intravenous endotoxin in humans: evaluation of a new clinical lot of Escherichia coli O:113 endotoxin. *J Infect Dis.* 1999;179(5):1278-1282. doi:10.1086/314717.
78. Capuron L, Gumnick JF, Musselman DL, et al. Neurobehavioral effects of interferon-alpha in cancer patients: phenomenology and paroxetine responsiveness of symptom dimensions. *Neuropsychopharmacology.* 2002;26(5):643-652.

79. Harrison NA, Brydon L, Walker C, et al. Neural origins of human sickness in interoceptive responses to inflammation. *Biol Psychiatry.* 2009;66(5):415-422. doi:10.1016/j.biopsych.2009.03.007.

80. Harrison NA, Brydon L, Walker C, Gray MA, Steptoe A, Critchley HD. Inflammation causes mood changes through alterations in subgenual cingulate activity and mesolimbic connectivity. *Biol Psychiatry.* 2009;66(5):407-414. doi:S0006-3223(09)00396-5 [pii] 10.1016/j.biopsych.2009.03.015.

81. Brydon L, Harrison NA, Walker C, Steptoe A, Critchley HD. Peripheral inflammation is associated with altered substantia nigra activity and psychomotor slowing in humans. *Biol Psychiatry.* 2008;63(11):1022-1029. doi:S0006-3223(07)01241-3 [pii] 10.1016/j.biopsych.2007.12.007.

82. Runyan CW, Johnson RM, Yang J, et al. Risk and protective factors for fires, burns, and carbon monoxide poisoning in US households. *Am J Prevent Med.* 2005;28(1):102-108.

83. O'Malley AS, Forrest CB. Beyond the examination room: primary care performance and the patient-physician relationship for low-income women. *J Gen Intern Med.* 2002;17(1):66-74. doi:10.1046/j.1525-1497.2002.10338.x.

84. Irwin MR, Cole S, Olmstead R, et al. Moderators for depressed mood and systemic and transcriptional inflammatory responses: a randomized controlled trial of endotoxin. *Neuropsychopharm.* 2019;44(3):635-641. doi:10.1038/s41386-018-0259-6.

85. Irwin MR, Olmstead R, Breen EC, et al. Cognitive behavioral therapy and tai chi reverse cellular and genomic markers of inflammation in late-life insomnia: a randomized controlled trial. *Biol Psychiatry.* 2015;78(10):721-729. doi:10.1016/j.biopsych.2015.01.010.

86. Black DS, O'Reilly GA, Olmstead R, Breen EC, Irwin MR. Mindfulness meditation and improvement in sleep quality and daytime impairment among older adults with sleep

disturbances: a randomized clinical trial. *JAMA internal medicine.* 2015;175(4):494-501. doi:10.1001/jamainternmed.2014.8081.

87. First MB, Spitzer RL, Gibbon M, Williams JB. Structured Clinical Interview for DSM-IV Axis I Disorders - Patient Edition, Version 2.0. In. New York, New York: New York State Psychiatric Institute; 1996.

88. Edinger J, Wyatt J, Olsen M. Reliability and validity of insomnia diagnoses derived from the Duke Structured Interview for Sleep Disorders. *Sleep.* 2009;32:A265.

89. Fernandez-Mendoza J, Baker JH, Vgontzas AN, Gaines J, Liao D, Bixler EO. Insomnia symptoms with objective short sleep duration are associated with systemic inflammation in adolescents. *Brain Behav Immun.* 2017;61:110-116. doi:10.1016/j.bbi.2016.12.026.

90. Vgontzas AN, Basta M, Fernandez-Mendoza J. Subjective short sleep duration: what does it mean? *Sleep Med Rev.* 2014;18(4):291-292. doi:10.1016/j.smrv.2014.04.002.

91. Vgontzas AN, Fernandez-Mendoza J, Liao D, Bixler EO. Insomnia with objective short sleep duration: the most biologically severe phenotype of the disorder. *Sleep Med Rev.* 2013;17(4):241-254. doi:10.1016/j.smrv.2012.09.005.

92. Weimin L, Rongguang W, Dongyan H, Xiaoli L, Wei J, Shiming Y. Assessment of a portable monitoring device WatchPAT 200 in the diagnosis of obstructive sleep apnea. *Eur Arch Otorhinolaryngol.* 2013;270(12):3099-3105. doi:10.1007/s00405-013-2555-4.

93. Bajpai S, Im KB, Dyken ME, Sodhi SK, Fiedorowicz JG. Obstructive sleep apnea and risk for late-life depression. *Ann Clin Psychiatry.* 2014;26(3):163-170.

94. O'Connor M-F, Bower JE, Cho HJ, et al. To assess, to control, to exclude: Effects of biobehavioral factors on circulating inflammatory markers. *Brain Behavior and Immunity.* 2009;23(7):887-897. doi:10.1016/j.bbi.2009.04.005.

95. Ishii S, Karlamangla AS, Bote M, et al. Gender, obesity and repeated elevation of C-reactive protein: data from the CARDIA cohort. *PLoS One.* 2012;7(4):e36062. doi:10.1371/journal.pone.0036062.

96. Buysse DJ, Reynolds CF, 3rd, Monk TH, Berman SR, Kupfer DJ. The Pittsburgh Sleep Quality Index: a new instrument for psychiatric practice and research. *Psychiatry Res.* 1989;28(2):193-213.

97. Irwin M, Artin KH, Oxman MN. Screening for depression in the older adult: criterion validity of the 10-item Center for Epidemiological Studies Depression Scale (CES-D). *Arch Intern Med.* 1999;159(15):1701-1704.

98. Drill R, Nakash O, DeFife JA, Westen D. Assessment of clinical information: Comparison of the validity of a Structured Clinical Interview (the SCID) and the Clinical Diagnostic Interview. *J Nerv Ment Dis.* 2015;203(6):459-462. doi:10.1097/NMD.0000000000000300.

99. Beloosesky Y, Weiss A, Mansur N. Validity of the Medication-based Disease Burden Index compared with the Charlson Comorbidity Index and the Cumulative Illness Rating Scale for geriatrics: a cohort study. *Drugs & aging.* 2011;28(12):1007-1014. doi:10.2165/11597040-000000000-00000.

100. Putnam KG, Buist DSM, Fishman PA, et al. Chronic disease score as a predictor of hospitilization. *Epidemiology.* 2002;13:340-346.

101. von Korff M, Wagner EH, Saunders K. A chronic disease score from automated pharmacy data. *J Clin Epidem.* 1992;45:197-203.

102. Folstein MF, Folstein SE, McHugh PR. "Mini-mental state". A practical method for grading the cognitive state of patients for the clinician. *J Psychiatr Res.* 1975;12(3):189-198.

103. Morin CM, Belleville G, Belanger L, Ivers H. The Insomnia Severity Index: psychometric indicators to detect insomnia cases and evaluate treatment response. *Sleep.* 2011;34(5):601-608.

104. Cole JC, Motivala SJ, Buysse DJ, Oxman MN, Levin MJ, Irwin MR. Validation of a 3-factor scoring model for the Pittsburgh sleep quality index in older adults. *Sleep.* 2006;29(1):112-116.

105. Monk TH, Reynolds CF, Kupfer DJ, et al. The Pittsburgh Sleep Diary. *J Sleep Res.* 1994;3(2):111-120. doi:jsr003002111 [pii].

106. Sharma SK, Vasudev C, Sinha S, Banga A, Pandey RM, Handa KK. Validation of the modified Berlin questionnaire to identify patients at risk for the obstructive sleep apnoea syndrome. *Indian J Med Res.* 2006;124(3):281-290.

107. Juda M, Vetter C, Roenneberg T. The Munich ChronoType Questionnaire for Shift-Workers (MCTQShift). *J Biol Rhythms.* 2013;28(2):130-140. doi:10.1177/0748730412475041.

108. Donovan KA, Stein KD, Lee M, Leach CR, Ilozumba O, Jacobsen PB. Systematic review of the multidimensional fatigue symptom inventory-short form. *Support Care Cancer.* 2015;23(1):191-212. doi:10.1007/s00520-014-2389-7.

109. Stein KD, Jacobsen PB, Blanchard CM, Thors C. Further validation of the multidimensional fatigue symptom inventory-short form. *J Pain Symptom Manage.* 2004;27(1):14-23. doi:10.1016/j.jpainsymman.2003.06.003.

110. Kroenke K, Spitzer RL, Williams JB. The PHQ-9: validity of a brief depression severity measure. *J Gen Intern Med.* 2001;16(9):606-613. doi:jgi01114 [pii].

111. Steer RA, Rissmiller DJ, Beck AT. Use of the Beck Depression Inventory-II with depressed geriatric inpatients. *Behaviour Research and Therapy.* 2000;38:311-318.

112. Steer RA, Beck AT. The Beck Depression Inventory-II. In: Craighead WE, Nemeroff CB, eds. *The Corsini encyclopedia of psychology and behavioral science.* Vol 1. 3rd ed. New York: Wiley; 2000:178-179.

113. Beck AT, Steer, R.A. *Manual for the Beck anxiety inventory.* San Antonio: Psychological Corporation; 1990.

114. Rutter LA, Brown TA. Psychometric Properties of the Generalized Anxiety Disorder Scale-7 (GAD-7) in Outpatients with Anxiety and Mood Disorders. *J Psychopathol Behav Assess.* 2017;39(1):140-146. doi:10.1007/s10862-016-9571-9.

115. Rush AJ, Giles DE, Schlesser MA, Fulton CL, Weissenburger J, Burns C. The Inventory for Depressive Symptomatology (IDS): preliminary findings. *Psychiatry research.* 1986;18(1):65-87.

116. Cutrona CE, Russell D. The provisions of social relationships and adaption to stress. I. In: Jones H, Pearlman D, eds. *Advances in personal relationship. a research manual.* Greenwich, CT: Jai Press Inc; 1987:37-67.

117. Fraley RC, Waller NG, Brennan KA. An item response theory analysis of self-report measures of adult attachment. *J Pers Soc Psychol.* 2000;78(2):350-365.

118. Cohen S, Hoberman H. Positive events and social supports as buffers of life change stress. *Journal of Applied Social Psychology.* 1983;13:99-125.

119. Cohen S, Kamarck T, Mermelstein R. A global measure of perceived stress. *J Health Soc Behav.* 1983;24(4):385-396.

120. Repetti RL, Taylor SE, Seeman TE. Risky families: family social environments and the mental and physical health of offspring. *Psychol Bull.* 2002;128(2):330-366.

121. Amireault S, Godin G, Lacombe J, Sabiston CM. Validation of the Godin-Shephard Leisure-Time Physical Activity Questionnaire classification coding system using accelerometer assessment among breast cancer survivors. *Journal of cancer survivorship : research and practice.* 2015;9(3):532-540. doi:10.1007/s11764-015-0430-6.

122. McHorney CA, Ware JE, Jr., Raczek AE. The MOS 36-Item Short-Form Health Survey (SF-36): II. Psychometric and clinical tests of validity in measuring physical and mental health constructs. *Med Care.* 1993;31(3):247-263.

123. Shacham S. A shortened version of the Profile of Mood States. *J Pers Assess.* 1983;47(3):305-306.

124. Norcross JC, Guadagnoli E, Prochaska JO. Factor structure of the Profile of Mood States (POMS): two partial replications. *J Clin Psychol.* 1984;40(5):1270-1277.

125. McNair DM, Lorr M, Droppleman LF. *Manual for the Profile of Mood States.* San Diego: Educational and Industrial Testing Service; 1992.

126. Hammond MF. Rating depression severity in the elderly physically ill patient: reliability and factor structure of the Hamilton and the Montgomery-Asberg Depression Rating Scales. *International Journal of Geriatric Psychiatry.* 1998;13:257-261.

127. Moieni M, Muscatell KA, Jevtic I, Breen EC, Irwin MR, Eisenberger NI. Sex Differences in the Effect of Inflammation on Subjective Social Status: A Randomized Controlled Trial of Endotoxin in Healthy Young Adults. *Frontiers in psychology.* 2019;10:2167. doi:10.3389/fpsyg.2019.02167.

128. Brown C, Schulberg HC, Madonia MJ. Assessing depression in primary care practice with the Beck Depression Inventory and the Hamilton Rating Scale for Depression. *Psychological Assessment.* 1995;7:59-65.

129. Endicott J, Cohen J, Nee J, Fleiss J, Sarantakos S. Hamilton Depression Rating Scale. *Archives of General Psychiatry.* 1981;38:98-103.

130. Pollak SD, Messner M, Kistler DJ, Cohn JF. Development of perceptual expertise in emotion recognition. *Cognition.* 2009;110(2):242-247. doi:10.1016/j.cognition.2008.10.010.

131. Pollak SD, Sinha P. Effects of early experience on children's recognition of facial displays of emotion. *Dev Psychol.* 2002;38(5):784-791. doi:10.1037//0012-1649.38.5.784.

132. Piber D, Eisenberger NI, Olmstead R, et al. Sleep, inflammation, and perception of sad facial emotion: A laboratory-based study in older adults. *Brain Behav Immun.* 2020;89:159-167. doi:10.1016/j.bbi.2020.06.011.

133. van der Helm E, Gujar N, Walker MP. Sleep deprivation impairs the accurate recognition of human emotions. *Sleep.* 2010;33(3):335-342.

134. Tottenham N, Tanaka JW, Leon AC, et al. The NimStim set of facial expressions: judgments from untrained research participants. *Psychiatry Res.* 2009;168(3):242-249. doi:10.1016/j.psychres.2008.05.006.

135. Pizzagalli DA, Iosifescu D, Hallett LA, Ratner KG, Fava M. Reduced hedonic capacity in major depressive disorder: evidence from a probabilistic reward task. *J Psychiatr Res.* 2008;43(1):76-87. doi:10.1016/j.jpsychires.2008.03.001.

136. Vrieze E, Pizzagalli DA, Demyttenaere K, et al. Reduced reward learning predicts outcome in major depressive disorder. *Biol Psychiatry.* 2013;73(7):639-645. doi:10.1016/j.biopsych.2012.10.014.

137. Whitton AE, Treadway MT, Pizzagalli DA. Reward processing dysfunction in major depression, bipolar disorder and schizophrenia. *Curr Opin Psychiatry.* 2015;28(1):7-12. doi:10.1097/YCO.0000000000000122.

138. MacLeod C, Mathews A, Tata P. Attentional bias in emotional disorders. *J Abnorm Psychol.* 1986;95(1):15-20. doi:10.1037//0021-843x.95.1.15.

139. Weischer M, Nordestgaard BG, Cawthon RM, Freiberg JJ, Tybjaerg-Hansen A, Bojesen SE. Short telomere length, cancer survival, and cancer risk in 47102 individuals. *J Natl Cancer Inst.* 2013;105(7):459-468. doi:10.1093/jnci/djt016.

140. Bunney BG, Bunney WE. Mechanisms of rapid antidepressant effects of sleep deprivation therapy: clock genes and circadian rhythms. *Biol Psychiatry.* 2013;73(12):1164-1171. doi:10.1016/j.biopsych.2012.07.020.

141. Treadway MT, Buckholtz JW, Schwartzman AN, Lambert WE, Zald DH. Worth the 'EEfRT'? The effort expenditure for rewards task as an objective measure of motivation and anhedonia. *PLoS One.* 2009;4(8):e6598. doi:10.1371/journal.pone.0006598.

142. Boyle CC, Cho JH, Eisenberger NI, et al. Motivation and sensitivity to monetary reward in late-life insomnia: moderating role of sex and the inflammatory marker CRP. *Neuropsychopharmacology.* 2020;45(10):1664-1671. doi:10.1038/s41386-020-0735-7.

143. Snaith RP, Hamilton M, Morley S, Humayan A, Hargreaves D, Trigwell P. A scale for the assessment of hedonic tone the Snaith-Hamilton Pleasure Scale. *Br J Psychiatry.* 1995;167(1):99-103.

144. Ho PM, Cooper AJ, Hall PJ, Smillie LD. Factor structure and construct validity of the temporal experience of pleasure scales. *J Pers Assess.* 2015;97(2):200-208. doi:10.1080/00223891.2014.940625.

145. Inagaki TK, Muscatell KA, Moieni M, et al. Yearning for connection? Loneliness is associated with increased ventral striatum activity to close others. *Social cognitive and affective neuroscience.* 2016;11(7):1096-1101. doi:10.1093/scan/nsv076.

146. Inagaki TK, Muscatell KA, Irwin MR, et al. The role of the ventral striatum in inflammatory-induced approach toward support figures. *Brain Behav Immun.* 2015;44:247-252. doi:10.1016/j.bbi.2014.10.006.

147. Russell D, Peplau LA, Cutrona CE. The revised UCLA Loneliness Scale: concurrent and discriminant validity evidence. *J Pers Soc Psychol.* 1980;39(3):472-480.

148. Sarason IG, Levine HM, Basham RB. Assessing social support: The Social Support Questionnaire. *J Personality Soc Psychol.* 1983;44:127-139.

149. Shakespeare-Finch J, Obst PL. The development of the 2-Way Social Support Scale: a measure of giving and receiving emotional and instrumental support. *J Pers Assess.* 2011;93(5):483-490. doi:10.1080/00223891.2011.594124.

150. Bifulco A, Mahon J, Kwon JH, Moran PM, Jacobs C. The Vulnerable Attachment Style Questionnaire (VASQ): an interview-based measure of attachment styles that predict depressive disorder. *Psychol Med.* 2003;33(6):1099-1110. doi:10.1017/s0033291703008237.

151. Moieni M, Irwin MR, Jevtic I, Breen EC, Eisenberger NI. Inflammation impairs social cognitive processing: A randomized controlled trial of endotoxin. *Brain Behav Immun.* 2015;48:132-138. doi:10.1016/j.bbi.2015.03.002.

152. Adler NE, Epel ES, Castellazzo G, Ickovics JR. Relationship of subjective and objective social status with psychological and physiological functioning: preliminary data in healthy white women. *Health Psychol.* 2000;19(6):586-592. doi:10.1037//0278-6133.19.6.586.

153. Duke D, Krishnan M, Faith M, Storch EA. The psychometric properties of the Brief Fear of Negative Evaluation Scale. *J Anxiety Disord.* 2006;20(6):807-817. doi:10.1016/j.janxdis.2005.11.002.

154. Collins KA, Westra HA, Dozois DJ, Stewart SH. The validity of the brief version of the Fear of Negative Evaluation Scale. *J Anxiety Disord.* 2005;19(3):345-359. doi:10.1016/j.janxdis.2004.02.003.

155. Mehrabian A. Evidence bearing on the affiliative tendency (MAFF) andsensitivity to rejection (MSR) scales. *CurrPsychol.* 1994;13:97-116.

156. Hamilton DA, Johnson TE, Redhead ES, Verney SP. Control of rodent and human spatial navigation by room and apparatus cues. *Behavioural processes.* 2009;81(2):154-169. doi:10.1016/j.beproc.2008.12.003.

157. Skelton RW, Bukach CM, Laurance HE, Thomas KG, Jacobs JW. Humans with traumatic brain injuries show place-learning deficits in computer-generated virtual space. *J Clin Exp Neuropsychol.* 2000;22(2):157-175. doi:10.1076/1380-3395(200004)22:2;1-1;FT157.

158. Sicard V, Moore RD, Simard A, Lavoie G, Ellemberg D. Psychometric properties of a color-shape version of the switch task. *Appl Neuropsychol Adult.* 2022;29(5):1020-1029. doi:10.1080/23279095.2020.1842410.

159. Hutton SB, Ettinger U. The antisaccade task as a research tool in psychopathology: a critical review. *Psychophysiology.* 2006;43(3):302-313. doi:10.1111/j.1469-8986.2006.00403.x.

160. Petrowski K, Schmalbach B, Jagla M, Franke GH, Brahler E. Norm values and psychometric properties of the brief symptom inventory-18 regarding individuals between

the ages of 60 and 95. *BMC Med Res Methodol.* 2018;18(1):164. doi:10.1186/s12874-018-0631-6.

161. Fredrickson BL, Grewen KM, Algoe SB, et al. Psychological well-being and the human conserved transcriptional response to adversity. *PLoS One.* 2015;10(3):e0121839. doi:10.1371/journal.pone.0121839.

162. Cole SW, Levine ME, Arevalo JM, Ma J, Weir DR, Crimmins EM. Loneliness, eudaimonia, and the human conserved transcriptional response to adversity. *Psychoneuroendocrinology.* 2015;62:11-17. doi:10.1016/j.psyneuen.2015.07.001.

163. Cho JH, Irwin MR, Eisenberger NI, Lamkin DM, Cole SW. Transcriptomic predictors of inflammation-induced depressed mood. *Neuropsychopharmacology.* 2019;44(5):923-929. doi:10.1038/s41386-019-0316-9.

164. Eisenberger NI, Moieni M, Inagaki TK, Muscatell KA, Irwin MR. In Sickness and in Health: The Co-Regulation of Inflammation and Social Behavior. *Neuropsychopharmacology.* 2017;42(1):242-253. doi:10.1038/npp.2016.141.

165. Preacher KJ, Hayes AF. SPSS and SAS procedures for estimating indirect effects in simple mediation models. *Behav Res Methods Instrum Comput.* 2004;36(4):717-731.

166. Hofmann SG, Curtiss JE, Hayes SC. Beyond linear mediation: Toward a dynamic network approach to study treatment processes. *Clin Psychol Rev.* 2020;76:101824. doi:10.1016/j.cpr.2020.101824.

167. Hayes AF. An Index and Test of Linear Moderated Mediation. *Multivariate Behav Res.* 2015;50(1):1-22. doi:10.1080/00273171.2014.962683.

168. Hayes AF, Preacher KJ. Quantifying and Testing Indirect Effects in Simple Mediation Models When the Constituent Paths Are Nonlinear. *Multivariate Behav Res.* 2010;45(4):627-660. doi:10.1080/00273171.2010.498290.

169. Hayes AF. *Introduction to mediation, moderation, and conditional process analysis: A regression-based approach.*: Guilford Press; 2022.
