# Supplementary material for: Inflammatory Exposure and Depression in Older Adults With Insomnia: A Randomized Clinical Trial
Source: JAMA Psychiatry. 2025 Jul 16;82(9):859–67. doi: 10.1001/jamapsychiatry.2025.1327 (PMC12268530; doi:10.1001/jamapsychiatry.2025.1327)
Supplement: Supplement 2. — eMethods. Assessment of Inflammatory Markers eResults. Supplementary Results eTable 1. Characteristics of Participants, by Group and Conditiona eTable 2. Linear Mixed Model Indices for the Primary Outcome, POMS-D eFigure 1. Changes in Inflammatory Cytokines, Secondary Outcome eFigure 2. Changes in Feelings of Social Disconnection, Secondary Outcome eFigure 3. Changes in MADRS Anhedonia, Secondary Outcome eFigure 4. Association Between Depressed Mood and the Inflammatory Composite in the Insomnia Group and Controls eReference. [file jamapsychiatry-e251327-s002.pdf]

Supplemental Online Content

Irwin MR, Boyle CC, Cho JH, et al. Inflammatory exposure and depression in older adults with insomnia: a randomized clinical trial. *JAMA Psychiatry*. Published online July 16, 2025.
doi:10.1001/jamapsychiatry.2025.1327

eMethods. Assessment of Inflammatory Markers

eResults. Supplementary Results

eTable 1. Characteristics of Participants, by Group and Condition[a]

eTable 2. Linear Mixed Model Indices for the Primary Outcome, POMS-D

eFigure 1. Changes in Inflammatory Cytokines, Secondary Outcome

eFigure 2. Changes in Feelings of Social Disconnection, Secondary Outcome

eFigure 3. Changes in MADRS Anhedonia, Secondary Outcome

eFigure 4. Association Between Depressed Mood and the Inflammatory Composite in the Insomnia Group and Controls

eReference.

This supplemental material has been provided by the authors to give readers additional information about their work.

© 2025 Irwin MR et al. *JAMA Psychiatry*.

eMethods. Assessment of Inflammatory Markers

Assessment of IL-6 and TNF

Plasma levels of inflammatory were assayed using the Meso Scale Discovery (MSD) MULTI-SPOT Assay System (Rockville, MD) employing a custom 5-plex from the Proinflammatory Panel 1 Human Kit. All samples were assayed in duplicate as a single batch by a single operator. In addition to IL-6 and TNF, MSD assays also evaluated plasma levels of IL-8, IL-10 and interferon-γ; results from these assays will be reported separately, as the aim of this study is to examine whether endotoxin induces increases in IL-6 and TNF in older adults, as has been found in adult samples,[1] and whether baseline levels of IL-6 and TNF and their response to endotoxin differ between those with insomnia as compared to older adult controls. Further, there are limited data showing that these additional cytokines relate to depressed mood in response to endotoxin; hence analyses of these additional cytokines would be exploratory, and beyond the scope of the present report.

Briefly, blood samples were repeatedly collected over the 9 hour protocol (12 hours in a subgroup; N=20), placed in EDTA tubes on ice, and processed within one hour at 4 °C to obtain plasma aliquots. Plasma aliquots were stored at −80 °C until assays were performed. All assays were performed according to the manufacturer's protocol. ECL signals were measured on the MESO QuickPlex SQ 120 instrument (Rockville, MD), and the DISCOVERY WORKBENCH software (Rockville, MD) was used to generate a 4-parameter logistic fit curve. For this study, quality control measures were obtained, and the mean intra-assay coefficient of variation (CV) for IL-6 was 3.25% with a range from 3.1% to 4.9%, and for TNF the mean was 4.1% with range from 2.6% to 5.7%. The mean inter-assay CV for IL-6 was 8.5% and for TNF was 7.6%.

© 2025 Irwin MR et al. *JAMA Psychiatry*.

eResults. Supplementary Results

Baseline characteristics of participants by condition and group

eTable 1 provides the baseline characteristics of the participants by condition and group.

Inflammatory outcomes

Changes in the inflammatory cytokines, IL-6 and TFN in response to endotoxin vs. placebo over the protocol are shown from the two groups (eFigure 1).

Exploratory analyses in subgroup prior to protocol modification

As noted, the duration of the protocol was modified from 12- to 9 hours once the safety of endotoxin in older adults was confirmed.

In the subgroup (N=20) who were evaluated for the 12 hour period, we found that levels of IL-6 and TNF remained elevated as compared to baseline, although differences were small. IL-6 and TNF levels continued to decrease from $T_{8.5}$ to T_{13} in this subgroup. Time comparisons between T_0 and $T_{13,}$ showed the following differences (baseline vs. T_{13}) for each cytokine concentration expressed as log transformed pg/ml in the overall sample (IL-6, 2.13 vs 2.57; P<0.001; TNF, 0.61 vs. 1.33; P<0.001; composite IL-6 + TNF, 2.74 vs. 3.90; P<0.001).

In the subgroup (N=20) who were evaluated for 12 hours prior to protocol modification, POMS-D scores continued to decrease. Time comparisons between T_0 and $T_{13,}$ showed similar POMS-D scores (overall sample, 0.51 vs. 0.60, P=0.79; insomnia, 0.37 vs 0.90, P=0.16; control, 0.57 vs 0.48, P=0.82).

Exploratory depression related outcomes: social disconnection and anhedonia

Endotoxin, as compared to placebo, induced increases of feeling of social disconnection (condition, $F_{10,1463}$ = 6.1; P<0.001), with greater increases in insomnia vs. control (condition x group, $F_{10,1463}$ = 2.3; P<0.01; eFigure 2), with similar effects covarying for baseline (condition x

© 2025 Irwin MR et al. *JAMA Psychiatry*.

group, $F_{10,1473}$ = 2.3; P=0.01). In insomnia, endotoxin induced greater feelings of social disconnection as compared to placebo (mean difference area under the curve, MD-AUC, 3.3; 95%CI, 1.08 – 5.57; $T_{0.5}$; P<0.05; $T_{1\text{-}5;}$ all P's<0.001; $T_{6\text{-}7}$; all P's<0.05), with no effect in control (MD-AUC, 0.36; 95% CI -0.99 – 1.71; all P's>0.5; eFigure 2).

Endotoxin, as compared to placebo, induced increases in the MADRS - anhedonia subscale (condition, F3,451 = 11.8; P<0.001), with greater increases in insomnia vs. control (condition x group, F3,451 = 2.9; P<0.05; eFigure)3, and similar effects adjusting for baseline (condition x group, F3,451 = 2.9; P<0.05). In insomnia, endotoxin induced greater increases in anhedonia as compared to placebo at all timepoints (MD-AUC, 5.50; 95%CI 0.89 – 10.10; T2, T4.5, P<0.001; T6.5, P<0.01), with effect in control limited to two timepoints (MD-AUC, 2.97; 95%CI 1.23 – 4.71; T2, P<.01;T6.5, P<0.05; eFigure 3).

© 2025 Irwin MR et al. *JAMA Psychiatry*.

eTable 1. Characteristics of Participants, by Group and Condition[a]

Characteristic	Controls		Insomnia	
	Endotoxin (N=53)	Placebo (N=54)	Endotoxin (N=26)	Placebo (N=27)
Age, mean (SD), y	65.8 ± 4.6	65.9 ± 4.3	65.8 ± 4.8	66.2 ± 5.3
Sex				
Female	23 (43.4)	30 (55.6)	17 (65.4)	14 (51.9)
Male	30 (56.6)	24 (44.4)	9 (34.6)	13 (48.1)
Race or ethnic group				
African American/Black	3 (5.7)	6 (11.1)	3 (11.5)	7 (25.9)
Asian	3 (5.7)	7 (13.0)	1 (3.8)	1 (3.7)
Multiracial	3 (5.7)	2 (3.7)	0 (0.0)	0 (0.0)
Pacific Islander	0 (0.0)	2 (3.7)	0 (0.0)	1 (3.7)
White	44 (83.0)	37 (68.5)	22 (84.6)	18 (66.7)
Ethnicity				
Hispanic/Latino	3 (5.7)	4 (7.4)	2 (7.7)	2 (7.4)
Non-Hispanic/Non-Latino	50 (94.3)	50 (92.6)	24 (92.3)	25 (92.6)
Marital status				
Married/Partnered	29 (45.3)	18 (33.3)	9 (34.6)	15 (55.6)
Income, mean (SD), $ in thousands	90.3 ± 56.4	91.6 ± 58.0	92.1 ± 48.2	81.4 ± 55.5
Fulltime employment	38 (71.7)	39 (72.2)	18 (69.2)	21 (77.8)
Education level, mean (SD), y	16.5 ± 2.4	17.1 ± 2.2	16.0 ± 1.3	16.1 ± 1.7
BMI, mean (SD)	25.1 ± 3.0	24.7 ± 3.1	25.2 ± 3.7	25.2 ± 3.6
Charlson Co-Morbidity Index, mean (SD)[b]	2.1 ± 0.4	2.2 ± 0.5	2.3 ± 0.6	2.4 ± 1.4

© 2025 Irwin MR et al. *JAMA Psychiatry*.

Sleep disturbance				
Duration of insomnia, mean (SD), y	N/A	NA	12.3 ± 10.2	14.4 ± 12.6
Insomnia Severity Index mean (SD)[c]	1.3 ± 2.0	1.3 ± 1.5	10.7 ± 5.4	10.9 ± 5.2
Use of hypnotic medications	0 (0.0)	0 (0.0)	1 (3.8)	0 (0.0)
Depression				
History of depression history[d]	8 (15.1)	6 (11.1)	10 (38.5)	5 (18.5)
Use of anti-depressants	0	0	0	0
PHQ-8 Score, mean (SD)[e]	0.4 ± 0.9	0.2 ± 0.4	2.7 ± 2.1	2.7 ± 3.2

Abbreviations: BMI, body mass index (calculated as weight in kilograms divided by height in meters squared; PHQ-8, Patient Health Questionnaire 8 (PHQ-9 without insomnia item)

[a.] Data are presented as number (percentage) unless otherwise indicated.
[b] The Charlson Co-Morbidity Index includes 17 categories of comorbidity, each with an assigned score of 1 to 6, depending on the risk of death associated with the condition; maximum score is 29
[c] Insomnia Severity Index rates severity of sleep disturbance according to DSM-5 criteria. Scores range from 0 to 28, with a score of <7 indicating no clinically significant insomnia.
[d] Lifetime history of depression is fulfilled by major depressive disorder in the Diagnostic and Statistical Manual--5 criteria, as determined following administration of the Structured Clinical Interview.
[e] PHQ-9 scores each of the 9 criteria for major depressive disorder in the DSM-IV-TR, with as "0" (not at all) to "3" (nearly every day); maximum score of 27. All eligible insomnia participants had sleep disturbance; hence, PHQ-8 scored each of the criteria for major depressive disorder with the exception of insomnia, yielding a maximum score of 24. Scores below 5 on the PH9-indicate none to minimal depression

© 2025 Irwin MR et al. *JAMA Psychiatry*.

eTable 2. Linear Mixed Model Indices for the Primary Outcome, POMS-D

Parameter*	Estimate	SE	df	t	P	95% CI
Intercept	3.933	1.592	154.7	2.47	0.015	0.788 - 7.078
[Group=HC]	-0.194	0.505	382.0	-0.38	0.71	-1.186 - 0.799
[Cond=Placebo]	-0.024	0.566	374.0	-0.04	0.97	-1.136 - 1.089
[Sex=Female]	0.652	0.675	423.7	0.97	0.34	-0.674 - 1.979
[Time=0]	0.047	0.350	1469.9	0.14	0.90	-0.639 - 0.733
[Time=0.5]	0.203	0.350	1469.9	0.58	0.57	-0.483 - 0.889
[Time=1]	1.629	0.350	1469.9	4.66	<.001	0.943 - 2.315
[Time=2]	1.928	0.356	1470.5	5.42	<.001	1.231 - 2.626
[Time=3]	1.089	0.350	1469.9	3.11	0.002	0.403 - 1.775
[Time=4]	0.192	0.350	1469.9	0.55	0.59	-0.494 - 0.878
[Time=5]	0.439	0.354	1469.0	1.24	0.22	-0.256 - 1.134
[Time=6]	0.572	0.350	1469.9	1.64	0.11	-0.114 - 1.258
[Time=7]	0.500	0.356	1470.5	1.41	0.16	-0.198 - 1.197
[Time=8]	0.141	0.361	1469.6	0.39	0.70	-0.567 - 0.848
[Group=HC] * [Cond=Placebo]	-0.357	0.721	384.3	-0.50	0.63	-1.773 - 1.060
[Group=HC] * [Sex=Female]	-0.467	0.801	412.6	-0.58	0.56	-2.042 - 1.107
[Time=0] * [Group=HC]	0.177	0.460	1469.7	0.38	0.71	-0.726 - 1.080
[Time=0.5] * [Group=HC]	-0.154	0.460	1469.7	-0.33	0.74	-1.056 - 0.749
[Time=1] * [Group=HC]	-1.218	0.460	1469.7	-2.65	0.008	-2.121 - -0.315
[Time=2] * [Group=HC]	-1.893	0.465	1470.1	-4.07	<.001	-2.804 - -0.981
[Time=3] * [Group=HC]	-0.788	0.460	1469.7	-1.71	0.09	-1.690 - 0.115
[Time=4] * [Group=HC]	-0.163	0.460	1469.7	-0.36	0.73	-1.066 - 0.740
[Time=5] * [Group=HC]	-0.615	0.464	1469.2	-1.33	0.19	-1.525 - 0.295
[Time=6] * [Group=HC]	-0.745	0.460	1469.7	-1.62	0.11	-1.648 - 0.158
[Time=7] * [Group=HC]	-0.592	0.467	1470.3	-1.27	0.21	-1.508 - 0.324
[Time=8] * [Group=HC]	-0.226	0.471	1469.3	-0.48	0.64	-1.149 - 0.697
[Cond=Placebo] * [Sex=Female]	-0.617	0.906	403.6	-0.68	0.50	-2.397 - 1.164
[Time=0] * [Cond=Placebo]	0.469	0.516	1469.4	0.91	0.37	-0.543 - 1.480
[Time=0.5] * [Cond=Placebo]	0.092	0.516	1469.4	0.18	0.86	-0.920 - 1.103
[Time=1] * [Cond=Placebo]	-1.768	0.516	1469.4	-3.43	<.001	-2.779 - -0.757
[Time=2] * [Cond=Placebo]	-1.955	0.520	1469.7	-3.76	<.001	-2.974 - -0.936
[Time=3] * [Cond=Placebo]	-1.012	0.516	1469.4	-1.96	0.05	-2.024 - -0.001
[Time=4] * [Cond=Placebo]	-0.037	0.516	1469.4	-0.07	0.94	-1.048 - 0.974

© 2025 Irwin MR et al. *JAMA Psychiatry*.

[Time=5] * [Cond=Placebo]	-0.622	0.519	1469.0	-1.20	0.24	-1.639 - 0.396
[Time=6] * [Cond=Placebo]	-0.065	0.516	1469.4	-0.13	0.90	-1.076 - 0.947
[Time=7] * [Cond=Placebo]	-0.338	0.525	1470.1	-0.64	0.52	-1.368 - 0.693
[Time=8] * [Cond=Placebo]	-0.115	0.523	1469.2	-0.22	0.83	-1.141 - 0.911
[Time=0] * [Sex=Female]	1.135	0.617	1470.8	1.84	0.07	-0.075 - 2.344
[Time=0.5] * [Sex=Female]	0.760	0.617	1470.8	1.23	0.22	-0.450 - 1.970
[Time=1] * [Sex=Female]	0.817	0.617	1470.8	1.32	0.19	-0.393 - 2.026
[Time=2] * [Sex=Female]	0.696	0.620	1471.0	1.12	0.27	-0.521 - 1.912
[Time=3] * [Sex=Female]	1.804	0.617	1470.8	2.92	0.004	0.594 - 3.014
[Time=4] * [Sex=Female]	2.272	0.617	1470.8	3.68	<.001	1.062 - 3.482
[Time=5] * [Sex=Female]	0.762	0.619	1470.5	1.23	0.22	-0.453 - 1.977
[Time=6] * [Sex=Female]	0.085	0.617	1470.8	0.14	0.90	-1.125 - 1.295
[Time=7] * [Sex=Female]	0.096	0.620	1471.1	0.16	0.88	-1.120 - 1.312
[Time=8] * [Sex=Female]	0.430	0.636	1471.7	0.68	0.50	-0.817 - 1.677
[Group=HC] * [Cond=Placebo] * [Sex=Female]	0.747	1.096	398.8	0.68	0.50	-1.408 - 2.902
[Time=0] * [Group=HC] * [Cond=Placebo]	-0.230	0.658	1469.7	-0.35	0.73	-1.520 - 1.060
[Time=0.5] * [Group=HC] * [Cond=Placebo]	0.388	0.658	1469.7	0.59	0.56	-0.903 - 1.678
[Time=1] * [Group=HC] * [Cond=Placebo]	1.576	0.658	1469.7	2.40	0.017	0.286 - 2.866
[Time=2] * [Group=HC] * [Cond=Placebo]	2.085	0.661	1469.9	3.16	0.002	0.789 - 3.381
[Time=3] * [Group=HC] * [Cond=Placebo]	0.988	0.659	1469.8	1.50	0.14	-0.304 - 2.280
[Time=4] * [Group=HC] * [Cond=Placebo]	0.049	0.659	1469.6	0.07	0.95	-1.243 - 1.341
[Time=5] * [Group=HC] * [Cond=Placebo]	0.912	0.660	1469.4	1.38	0.17	-0.383 - 2.207
[Time=6] * [Group=HC] * [Cond=Placebo]	0.271	0.659	1469.8	0.41	0.69	-1.021 - 1.563
[Time=7] * [Group=HC] * [Cond=Placebo]	0.633	0.667	1470.2	0.95	0.35	-0.676 - 1.941
[Time=8] * [Group=HC] * [Cond=Placebo]	0.243	0.667	1469.5	0.36	0.72	-1.065 - 1.551

© 2025 Irwin MR et al. *JAMA Psychiatry*.

[Time=0] * [Group=HC] * [Sex=Female]	-0.660	0.734	1471.0	-0.90	0.37	-2.100 - 0.779
[Time=0.5] * [Group=HC] * [Sex=Female]	-0.602	0.734	1471.0	-0.82	0.42	-2.042 - 0.837
[Time=1] * [Group=HC] * [Sex=Female]	-0.783	0.734	1470.4	-1.07	0.29	-2.223 - 0.658
[Time=2] * [Group=HC] * [Sex=Female]	-0.263	0.737	1470.5	-0.36	0.73	-1.709 - 1.183
[Time=3] * [Group=HC] * [Sex=Female]	-1.967	0.734	1470.4	-2.68	0.007	-3.408 - -0.527
[Time=4] * [Group=HC] * [Sex=Female]	-2.255	0.734	1470.4	-3.07	0.002	-3.695 - -0.814
[Time=5] * [Group=HC] * [Sex=Female]	-0.446	0.737	1470.2	-0.61	0.55	-1.891 - 0.998
[Time=6] * [Group=HC] * [Sex=Female]	0.028	0.734	1470.4	0.04	0.97	-1.412 - 1.469
[Time=7] * [Group=HC] * [Sex=Female]	-0.121	0.739	1470.6	-0.16	0.87	-1.570 - 1.328
[Time=8] * [Group=HC] * [Sex=Female]	-0.432	0.752	1471.0	-0.58	0.57	-1.906 - 1.042
[Time=0] * [Cond=Placebo] * [Sex=Female]	-1.463	0.828	1470.2	-1.77	0.08	-3.087 - 0.162
[Time=0.5] * [Cond=Placebo] * [Sex=Female]	-1.140	0.828	1470.2	-1.38	0.17	-2.764 - 0.484
[Time=1] * [Cond=Placebo] * [Sex=Female]	-0.551	0.828	1470.2	-0.67	0.51	-2.175 - 1.073
[Time=2] * [Cond=Placebo] * [Sex=Female]	-0.651	0.830	1470.3	-0.78	0.44	-2.280 - 0.978
[Time=3] * [Cond=Placebo] * [Sex=Female]	-2.350	0.828	1470.2	-2.84	0.005	-3.974 - -0.726
[Time=4] * [Cond=Placebo] * [Sex=Female]	-2.766	0.828	1470.2	-3.34	<.001	-4.390 - -1.142
[Time=5] * [Cond=Placebo] * [Sex=Female]	-0.966	0.830	1470.0	-1.16	0.25	-2.594 - 0.662
[Time=6] * [Cond=Placebo] * [Sex=Female]	-0.987	0.828	1470.2	-1.19	0.24	-2.611 - 0.637

© 2025 Irwin MR et al. *JAMA Psychiatry*.

[Time=7] * [Cond=Placebo] * [Sex=Female]	-0.234	0.838	1470.7	-0.28	0.78	-1.879 - 1.410
[Time=8] * [Cond=Placebo] * [Sex=Female]	-0.564	0.847	1470.9	-0.67	0.51	-2.225 - 1.096
[Time=0] * [Group=HC] * [Cond=Placebo] * [Sex=Female]	0.480	1.002	1470.4	0.48	0.64	-1.486 - 2.446
[Time=0.5] * [Group=HC] * [Cond=Placebo] * [Sex=Female]	0.409	1.002	1470.4	0.41	0.69	-1.557 - 2.375
[Time=1] * [Group=HC] * [Cond=Placebo] * [Sex=Female]	0.134	1.003	1470.0	0.13	0.90	-1.833 - 2.100
[Time=2] * [Group=HC] * [Cond=Placebo] * [Sex=Female]	0.116	1.005	1470.1	0.12	0.91	-1.855 - 2.086
[Time=3] * [Group=HC] * [Cond=Placebo] * [Sex=Female]	2.091	1.003	1470.0	2.08	0.037	0.123 - 4.059
[Time=4] * [Group=HC] * [Cond=Placebo] * [Sex=Female]	2.593	1.003	1470.0	2.59	0.01	0.625 - 4.561
[Time=5] * [Group=HC] * [Cond=Placebo] * [Sex=Female]	0.601	1.004	1469.9	0.60	0.55	-1.369 - 2.571
[Time=6] * [Group=HC] * [Cond=Placebo] * [Sex=Female]	0.698	1.003	1470.0	0.70	0.49	-1.270 - 2.666
[Time=7] * [Group=HC] * [Cond=Placebo] * [Sex=Female]	-0.054	1.017	1470.5	-0.05	0.96	-2.049 - 1.940
[Time=8] * [Group=HC] * [Cond=Placebo] * [Sex=Female]	0.374	1.021	1470.5	0.37	0.72	-1.629 - 2.377
TrueAge	-0.035	0.022	147.8	-1.63	0.11	-0.078 - 0.008
BMI	-0.037	0.032	147.9	-1.17	0.25	-0.100 - 0.026

* Parameters not listed are set to zero because they are redundant.

© 2025 Irwin MR et al. *JAMA Psychiatry*.

eFigure 1. Changes in Inflammatory Cytokines, Secondary Outcome

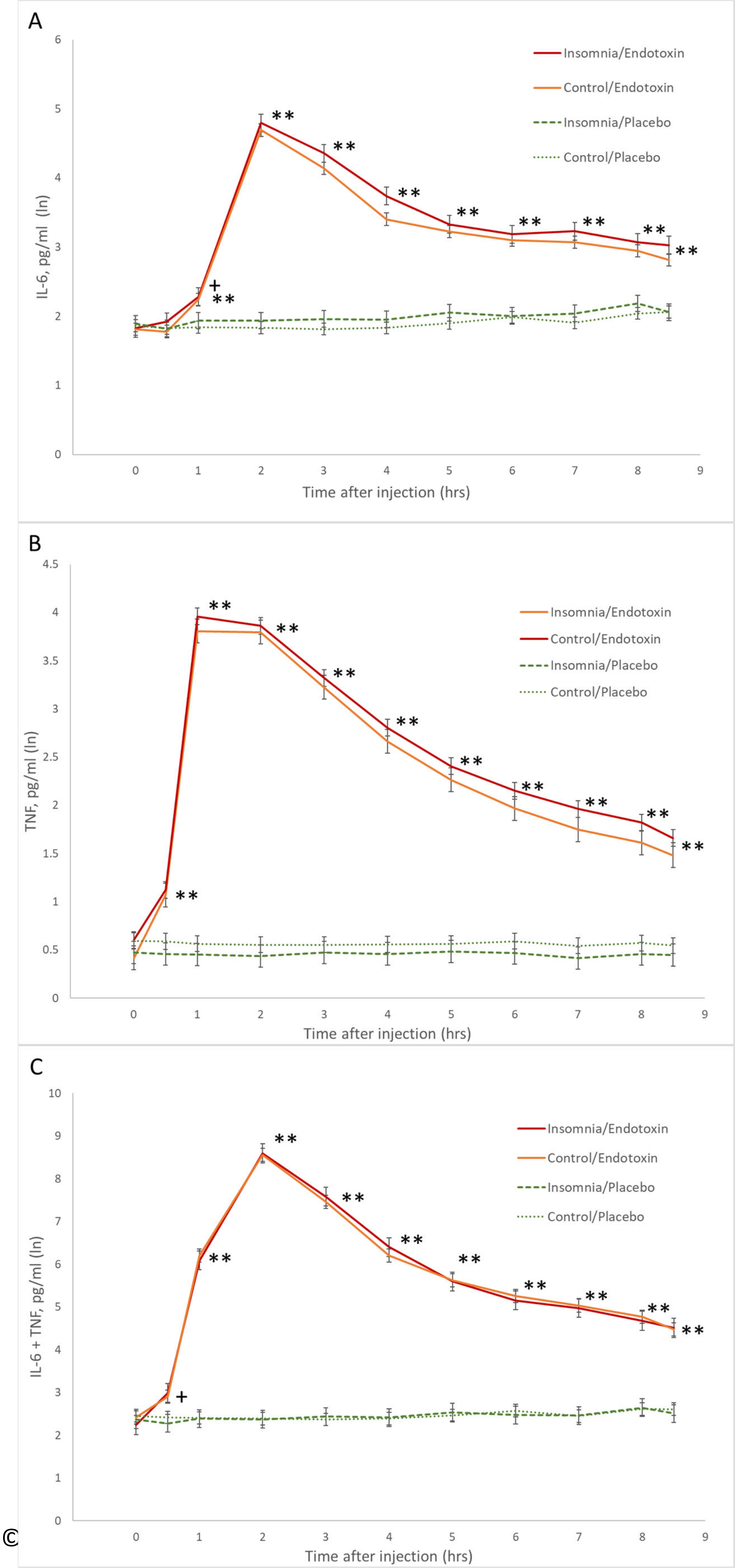

©

Changes over time in the endotoxin and placebo groups, stratified by insomnia status for (A) interleukin-6 (IL-6); (B) tumor necrosis factor (TNF); and (C) the composite of IL-6 and TNF. Circulating levels of inflammatory cytokines were assessed at baseline (T_0), 30 minutes after infusion ($T_{0.5}$) and then hourly following infusion for 8.5 hours ($T_{1-8.5}$). Timepoint condition differences (endotoxin vs. placebo) were tested in the insomnia and controls; significant differences indicated by, ** P<0.001; *P<0.01; +P<0;05.   Error bars depict the standard error of the mean.

© 2025 Irwin MR et al. *JAMA Psychiatry*.

eFigure 2. Changes in Feelings of Social Disconnection, Secondary Outcome

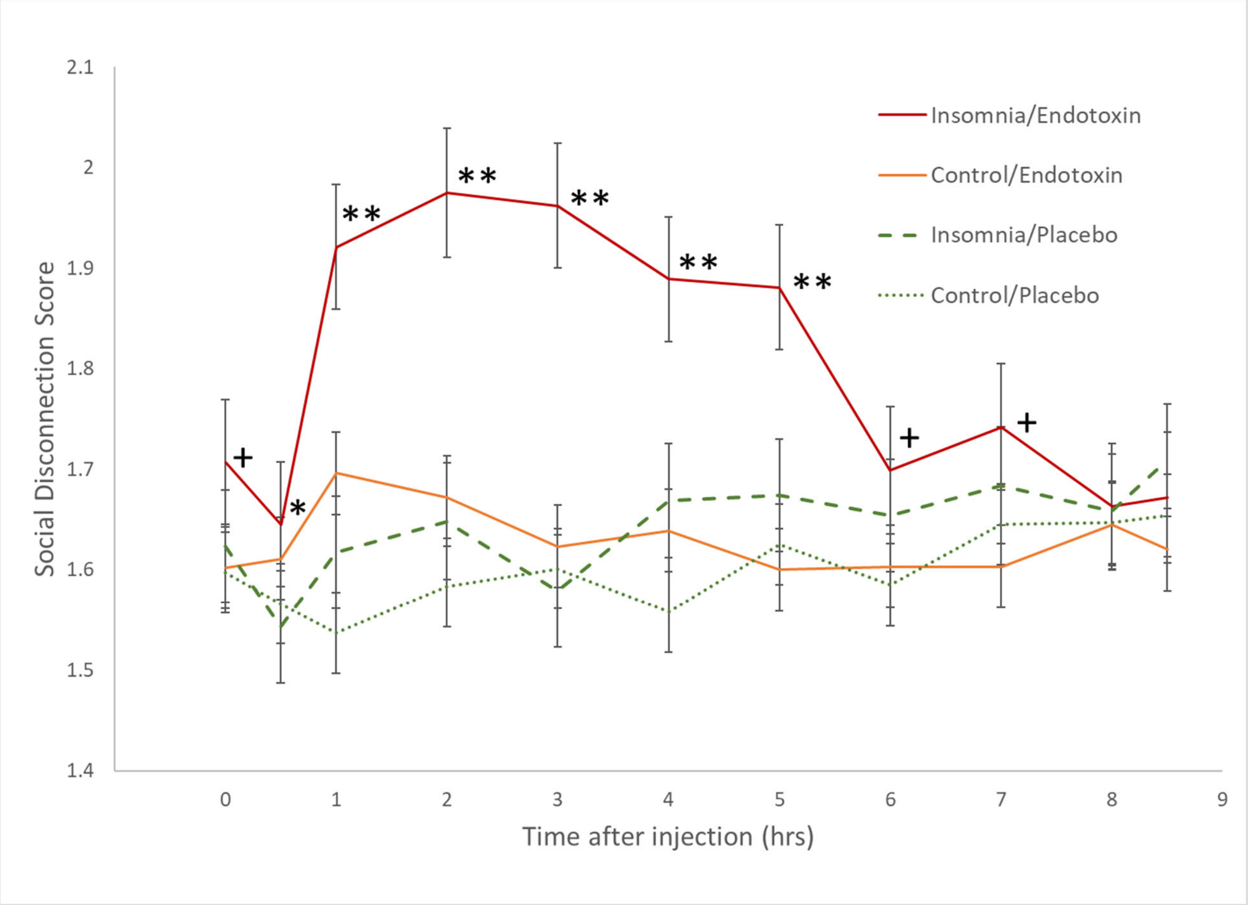

Changes over time in the endotoxin and placebo groups, stratified by insomnia status for feelings of social disconnection. Self-reported feelings of social disconnection were assessed at baseline (T_0), 30 minutes after infusion ($T_{0.5}$) and then hourly for the 8.5 hours ($T_{1\text{-}8.5}$). Timepoint condition differences (endotoxin vs. placebo) were tested in the insomnia and controls; significant differences indicated by, ** P<0.001; *P<0.01; +P<0;05. Error bars depict the standard error of the mean.

© 2025 Irwin MR et al. *JAMA Psychiatry*.

eFigure 3. Changes in MADRS Anhedonia, Secondary Outcome

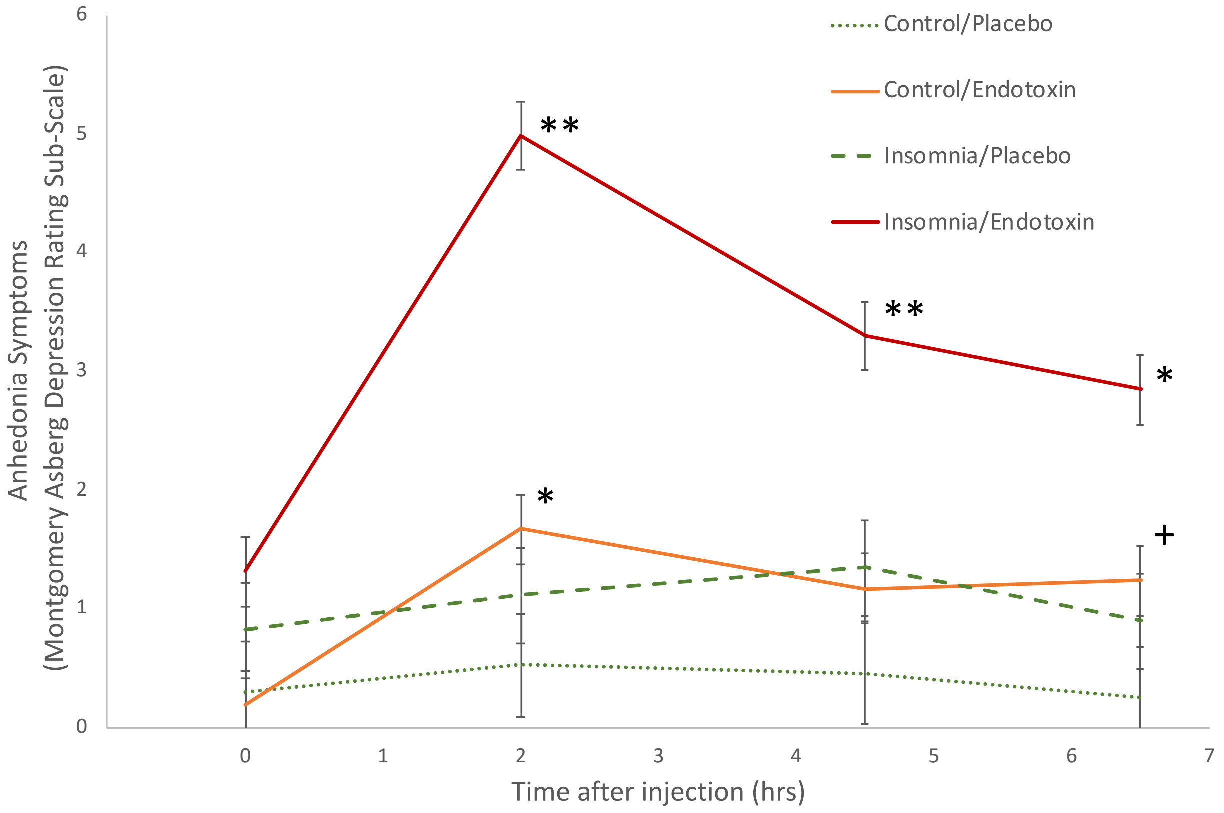

Changes over time in the endotoxin and placebo groups, stratified by insomnia status for feelings of social disconnection. Self-reported feelings of social disconnection were assessed at baseline (T_0), 30 minutes after infusion ($T_{0.5}$) and then hourly for the 8.5 hours ($T_{1\text{-}8.5}$). Timepoint condition differences (endotoxin vs. placebo) were tested in the insomnia and controls; significant differences indicated by, ** P<0.001; *P<0.01; +P<0;05. Error bars depict the standard error of the mean.

© 2025 Irwin MR et al. *JAMA Psychiatry*.

eFigure 4. Association Between Depressed Mood and the Inflammatory Composite in the Insomnia Group and Controls

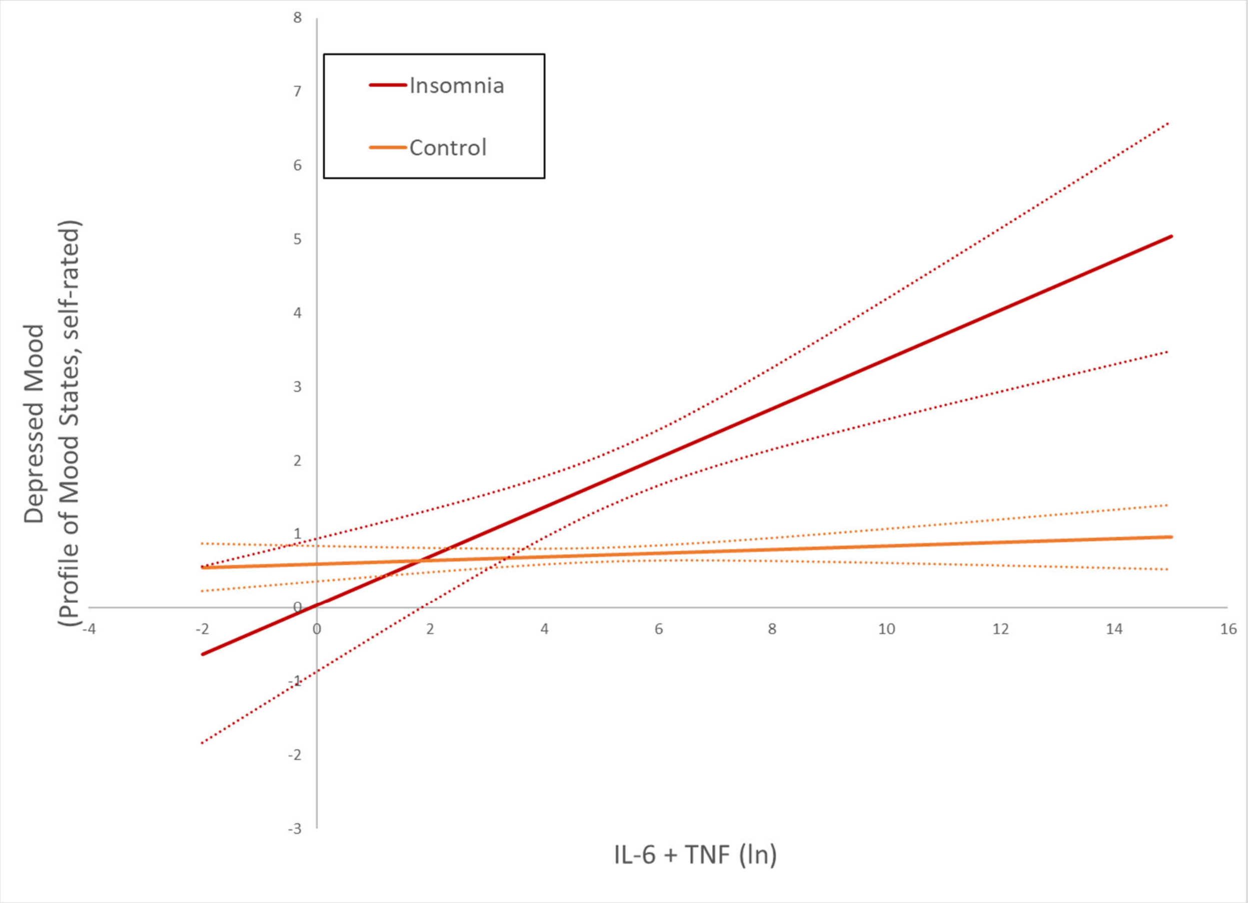

Shown are simple slopes with 95% confidence bands of the associations between depressed mood as assessed by POMS-D and the composite of IL-6 and TNF in older adults with insomnia, and those without insomnia (i.e., controls). The y-axis shows the POMS-D scores averaged over the duration of the protocol, and the x-axis shows the inflammatory composite averaged over the duration of the protocol. The broken lines illustrate the 95%CI for each group.

© 2025 Irwin MR et al. *JAMA Psychiatry*.

eReference

1. Moieni M, Irwin MR, Jevtic I, Olmstead R, Breen EC, Eisenberger NI. Sex differences in depressive and socioemotional responses to an inflammatory challenge: implications for sex differences in depression. *Neuropsychopharmacology.* 2015;40(7):1709-1716. doi:10.1038/npp.2015.17.

© 2025 Irwin MR et al. *JAMA Psychiatry*.
