# Supplementary material for: Inflammatory Exposure and Depression in Older Adults With Insomnia: A Randomized Clinical Trial
Source: JAMA Psychiatry. 2025 Jul 16;82(9):859–67. doi: 10.1001/jamapsychiatry.2025.1327 (PMC12268530; doi:10.1001/jamapsychiatry.2025.1327)
Supplement: Supplement 3. — Data Sharing Statement [file jamapsychiatry-e251327-s003.pdf]

Data Sharing Statement

Irwin. Inflammatory Exposure and Depression in Older Adults With Insomnia. *JAMA Psychiatry*. Published July 16, 2025. doi:10.1001/jamapsychiatry.2025.1327

Data
Additional Information: ClinicalTrials.gov NCT03256760
Data available: Yes
Data types: Deidentified participant data
How to access data: Request to be made to Michael R. Irwin, mirwin1@ucla.edu
When available: With publication

Supporting Documents
Document types: None

Additional Information
Who can access the data: Research whose proposed use of the data has been approved
Types of analyses: For an approved purpose
Mechanisms of data availability: After approval of proposal
